# A randomised controlled feasibility trial of E-health application supported care vs usual care after exacerbation of COPD: the RESCUE trial

**DOI:** 10.1038/s41746-020-00347-7

**Published:** 2020-10-30

**Authors:** Mal North, Simon Bourne, Ben Green, Anoop J. Chauhan, Tom Brown, Jonathan Winter, Tom Jones, Dan Neville, Alison Blythin, Alastair Watson, Matthew Johnson, David Culliford, Jack Elkes, Victoria Cornelius, Tom M. A. Wilkinson

**Affiliations:** 1 my mhealth Limited, Bournemouth, UK; 2 Portsmouth Hospitals NHS Trust, Portsmouth, UK; 3 NIHR ARC Wessex, University of Southampton, Southampton, UK; 4 University of Southampton Faculty of Medicine, Southampton, UK; 5 Imperial College London, London, UK

**Keywords:** Respiratory tract diseases, Disease prevention

## Abstract

Exacerbations of COPD are one of the commonest causes of admission and readmission to hospital. The role of digital interventions to support self-management in improving outcomes is uncertain. We conducted an open, randomised controlled trial of a digital health platform application (app) in 41 COPD patients recruited following hospital admission with an acute exacerbation. Subjects were randomised to either receive usual care, including a written self-management plan (*n* = 21), or the myCOPD app (*n* = 20) for 90 days. The primary efficacy outcome was recovery rate of symptoms measured by COPD assessment test (CAT) score. Exacerbations, readmission, inhaler technique quality of life and patient activation (PAM) scores were also captured by a blinded team. The app was acceptable in this care setting and was used by 17 of the 20 patients with sustained use over the study period. The treatment effect on the CAT score was 4.49 (95% CI: −8.41, −0.58) points lower in the myCOPD arm. Patients’ inhaler technique improved in the digital intervention arm (101 improving to 20 critical errors) compared to usual care (100 to 72 critical errors). Exacerbations tended to be less frequent in the digital arm compared to usual care; 18 vs 34 events. Hospital readmissions risk was numerically lower in the digital intervention arm: OR for readmission 0.383 (95% CI: 0.074, 1.987; *n* = 35). In this feasibility study of the digital self-management platform myCOPD, the app has proven acceptable to patients to use and use has improved exacerbation recovery rates, with strong signals of lower re-exacerbation and readmission rates over 90 days. myCOPD reduced the number of critical errors in inhaler technique compared to usual care with written self-management. This provides a strong basis for further exploration of the use of app interventions in the context of recently hospitalised patients with COPD and informs the potential design of a large multi-centre trial.

## Introduction

Chronic obstructive pulmonary disease (COPD) is one of the leading causes of hospital admission in the UK^1^. This creates a great burden on health services, especially during the winter months^2–5^. In the UK alone over 140,000 admissions occur on an annual basis driving over 1 million bed days, which is a significant contribution to NHS cost^1,6^. Despite improving approaches to acute management, COPD patients are often readmitted to hospital within weeks of discharge^7^. The UK RCP COPD audit identified that readmission rates were as high as 43% within 3 months of hospital discharge for patients with acute exacerbations^8^. This rate has risen significantly in recent years and interventions to prevent readmissions are desperately needed.

Exacerbation events are managed in hospital where care focuses on the rapid return of patients to their home environment once the acute crisis is over. Consequently, there is little time for clinicians to address the underlying drivers to exacerbations which, contrary to prior understanding, are both predictable and modifiable^9^. COPD self-management programmes have been demonstrated to reduce secondary care admission and health care usage^10–12^, and some have also demonstrated positive heath economic benefits^13^. Despite the virtues of implementing self-management in COPD patient populations, a report from the British lung Foundation suggests patients are not receiving the right support and advice from their clinical teams to be able to self-care effectively^14^. Clinical resource constraints exacerbate this situation and new, scalable and affordable models of care are required.

Recognition of the need to improve outcomes following admission for exacerbation of COPD has led to the development of discharge bundles and new pathways of care^15^. Core to these new strategies is support for self-management to improve a patient’s confidence, skills and knowledge essentials. Written self-management plans, review of inhaler technique and onward referral for exercise interventions are core to these strategies. However, the current model of delivery of these complex interventions relies on short face-to-face interactions between patients and specialist respiratory staff. Despite these innovations, the readmission rate for COPD is rising in the UK^8^ and new approaches to support patients to self-manage more effectively are required.

Digital technologies are now widely used by the population in general and to help support better health through behaviour change, including smoking cessation, activity and weight loss. The current evidence base around digital health interventions in chronic disease management is growing and there does appear to be the potential to improve health outcomes in a cost-effective and scalable manner^16,17^; the potential of digital health interventions for the treatment of chronic COPD has recently been reviewed^18,19^. Established digital tools have already been shown to effectively support exercise programmes in COPD^20–23^. A study by Rassouli et al further demonstrated the feasibility and acceptance of a digital pulmonary rehab intervention^24^. Furthermore, digital interventions to promote self-management support in primary and outpatient care settings have now been developed^25^. We hypothesised that digital interventions could also be used to improve disease understanding, treatment adherence and hence clinical outcomes following hospitalisation.

The digital application (app) myCOPD was developed by a multidisciplinary team of respiratory clinicians and people living with COPD. myCOPD is set apart from other digital interventions as it is the only multi-faceted online self-management app platform comprising of education programmes, a 6 week, gated, online pulmonary rehabilitation programme, inhaler technique videos and environmental alerts of weather and pollution. It was designed to support patients to self-manage effectively and provides a clinician interface to enable patients to be remotely monitored by their healthcare team^26^. We conducted a single-blinded, randomised controlled trial to explore the acceptability and feasibility of using this digital intervention to assist self-management in a vulnerable population of patients recently admitted to hospital with an acute exacerbation of COPD at a single NHS site in the UK. We sought to explore the safety and efficacy of the app compared with the standard of care with written self-management advice.

## Results

### Patients

**Fig. 1:**
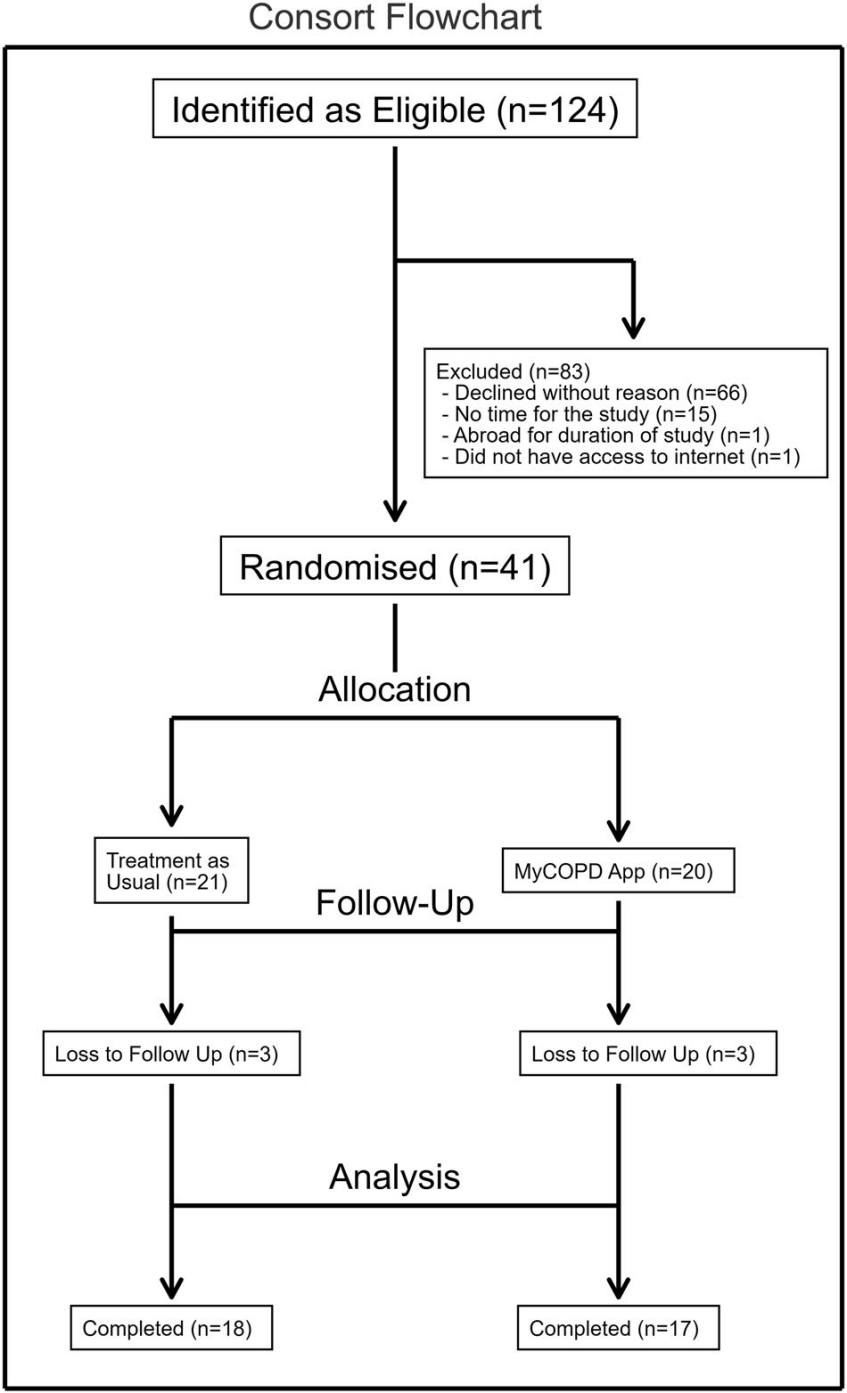
Flow diagram of study participants. Indicated is the consort flowchart of study participants through the study. Showing the number of eligible patients, number excluded and reasons for exclusion, number randomised to each arm, number of loses to follow up in each arm and number included in the analysis for each arm.

A total of 905 patients were admitted with respiratory presentation between June 2015 and December 2015. However, only 124 were bona fide COPD exacerbations making them eligible to participate. Factors including the diagnosis of other respiratory diseases such as asthma, a sensitivity or allergy to saccharin, lack of digital literacy or equipment and patient choice contributed to large numbers of patient ineligibility. Forty one (33%) patients were consented to enter the study and were randomised to one of the two study arms during the 6-month recruitment window. Of the 41 participants, 21 were randomised to Treatment as Usual (TAU) and 20 to the myCOPD app, patient flow is summarised in Fig. 1 and included subjects described in Table 1.

**Table 1:** Baseline participant characteristics.

Characteristics	Total *n* = 41		Treatment groups			
			TAU (*n* = 21)		myCOPD App (*n* = 20)	
Age, years	66.6	(7.0)	68.1	(7.4)	65.1	(6.3)
Sex						
Female	17	(41%)	10	(48%)	7	(35%)
Male	24	(59%)	11	(52%)	13	(65%)
COPD severity						
Moderate	14	(34%)	10	(48%)	4	(20%)
Severe	17	(41%)	6	(29%)	11	(55%)
Very severe	10	(24%)	5	(24%)	5	(25%)
Smoking status						
Current	12	(29%)	5	(24%)	7	(35%)
Ex	29	(71%)	16	(76%)	13	(65%)
Pack years	56.1	(36.0)	59.9	(32.5)	52.2	(39.8)
FEV1	1.2	(0.6)	1.2	(0.6)	1.2	(0.5)
FEV1 % predicted	44.1	(17.6)	46.5	(17.8)	41.5	(17.5)
FVC	2.5	(0.9)	2.6	(0.9)	2.5	(1.0)
FVC % predicted	75.8	(21.8)	80.1	(21.0)	71.1	(22.3)
COPD assessment test (CAT) score	27.0	(7.3)	28.0	(5.8)	26.0	(8.5)
Patient activation measure (PAM)	56.8	(11.5)	54.0	(11.2)	59.7	(11.4)
Modified MRC scale for dyspnoea	3.0	(1.2)	3.1	(1.1)	2.9	(1.3)
St Georges respiratory questionnaire (SGRQ)	67.3	(15.0)	68.1	(13.7)	66.4	(16.6)
Hospital anxiety and depression scale (HAD)	18.5	(8.5)	18.1	(6.1)	18.9	(10.6)
Work productivity activity impairment questionnaire (WPAI)	7.1	(2.2)	6.9	(2.3)	7.3	(2.0)
Veterans specific activity questionnaire (VSAQ) METs	1.9	(1.9)	1.7	(0.8)	2.2	(2.6)
Veterans specific activity questionnaire (VSAQ) score	2.9	(2.0)	2.6	(1.1)	3.2	(2.7)
Number of recorded exacerbations	3.1	(1.8)	3.2	(2.0)	2.9	(1.6)
Total number of errors	5.0	(3.2)	5.0	(3.3)	5.1	(3.1)

Data are *n* (%) or mean (SD).

Full data at all timepoints were collected for 35 participants, 18 (86%) in the TAU arm and 17 (85%) in the myCOPD arm. Six participants withdrew from the study with 3 from each treatment arm, 4 of these withdrew prior to month 1 and 2 prior to month 3, see Table 2. Four participants had missing data for the COPD assessment test (CAT) and 3 patients were missing the Patient Activated Measures scores.

**Table 2:** Feasibility outcomes.

Feasibility outcome	Total		TAU		MyCOPD	
	*n* = 41	%	*n* = 21	%	*n* = 20	%
Retention, *n*%	35	85	18	85	17	85
Withdrawal, *n*%	6	15	3	14	3	15
Days app used/week, mean (SD)					4.8	0.58
App used at minimum recommendation, *n*%					8	40
Incomplete data, *n*%	4	10	2	10	2	10

Retention is defined as participants who completed the study, data collected at 90 days.

The randomised cohort had a mean age of 66.6 years (SD 7.0), with the mean pack years of smoking being 56.1 (SD 36.0). There were 17 (41%) females in the study with the majority of participants having their COPD recorded as moderate (34%) or severe (41%). The mean CAT score at baseline was 27.0 (SD 7.3). Both adverse events (AEs) and serious adverse events (SAEs) were infrequently reported with 2 SAEs in myCOPD and 1 in TAU, see Table 3.

**Table 3:** Safety-adverse and severe adverse events.

	TAU (*n* = 21)	mHealth App (*n* = 20)
Adverse event		
Constipation		1
Serious adverse event		
Respiratory Infection other than AECOPD	1	
Constipation		1
Medication side effect		1

Titles of adverse event and serious event are given in bold.

These data are displayed due to its relevance to AEs and SAEs.

### App usage

**Fig. 2:**
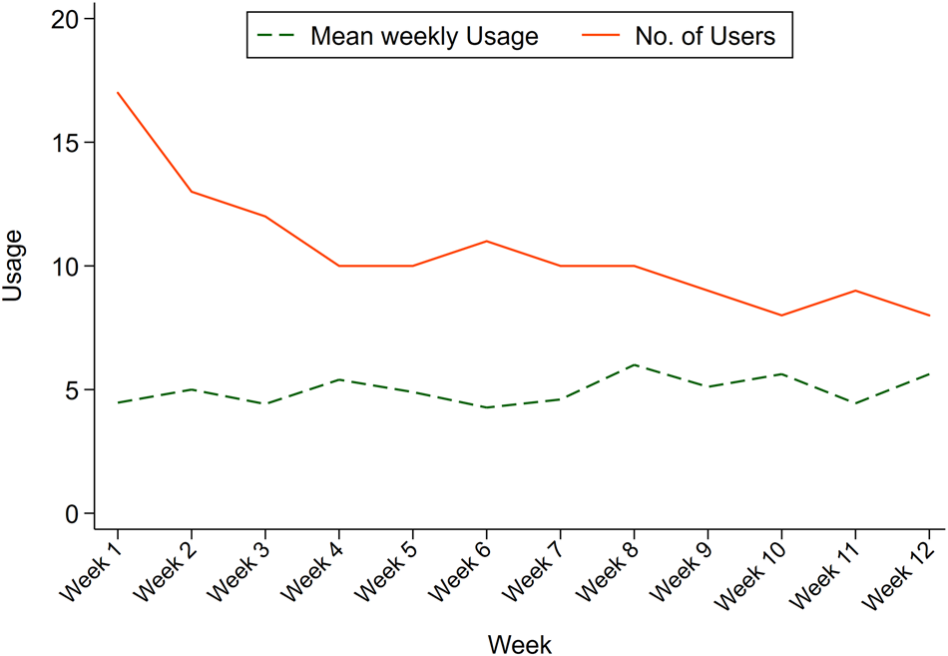
Mean weekly usage and mean number of users by week of the trial. Indicated is the mean weekly usage (green) and number of users of the app (red) over the 12 week study period. Mean weekly usage is the number of days per week that the app was accessed. The number of users is the number of participants that used the app at least once in that week.

Patients randomised to myCOPD had good engagement and, of the 20 participants randomised to myCOPD, 17 (85%) participants activated the app, all in the first week. The proportion of users was highest in the first week and lowest in the last week of the study with 8 (40%) users, Table 4. Weekly usage was 4.9 days, which did not significantly change for the duration of the study. Highest weekly usage occurred in week 8, with 10 (50%) users accessing the app for 6 of the 7 days. Lowest weekly usage occurred in week 6, with 11 (55%) users on average accessing the app for 4.2 days. Although days of app usage per week did not significantly, change there was a continual decline in the number of users per week, Fig. 2. At least 8 (40%) participants used the app once a week, minimum recommendation, for the whole duration of the trial. Of the 17 participants who activated the app, only 4 (24%) participants used it for the first week only.

**Table 4:** App usage and mean days used for the MyCOPD arm in participants who did not withdraw from the study.

Week of Trial	Total (*N* = 20)	
	Users, *n* %	Days used, mean (SD)
Week 1	17 (85%)	4.5 (2.37)
Week 2	13 (65%)	5 (1.83)
Week 3	12 (60%)	4.4 (2.39)
Week 4	10 (50%)	5.4 (1.78)
Week 5	10 (50%)	4.9 (1.91)
Week 6	11 (55%)	4.3 (2.20)
Week 7	10 (50%)	4.6 (2.12)
Week 8	10 (50%)	6 (1.33)
Week 9	9 (45%)	5.1 (2.09)
Week 10	8 (40%)	5.6 (1.77)
Week 11	9 (45%)	4.4 (2.65)
Week 12	8 (40%)	5.6 (2.13)

Users are participants who accessed the app on at least one day in the week under evaluation. This demonstrates the minimum amount of participant usage.

### Symptom control CAT

**Fig. 3:**
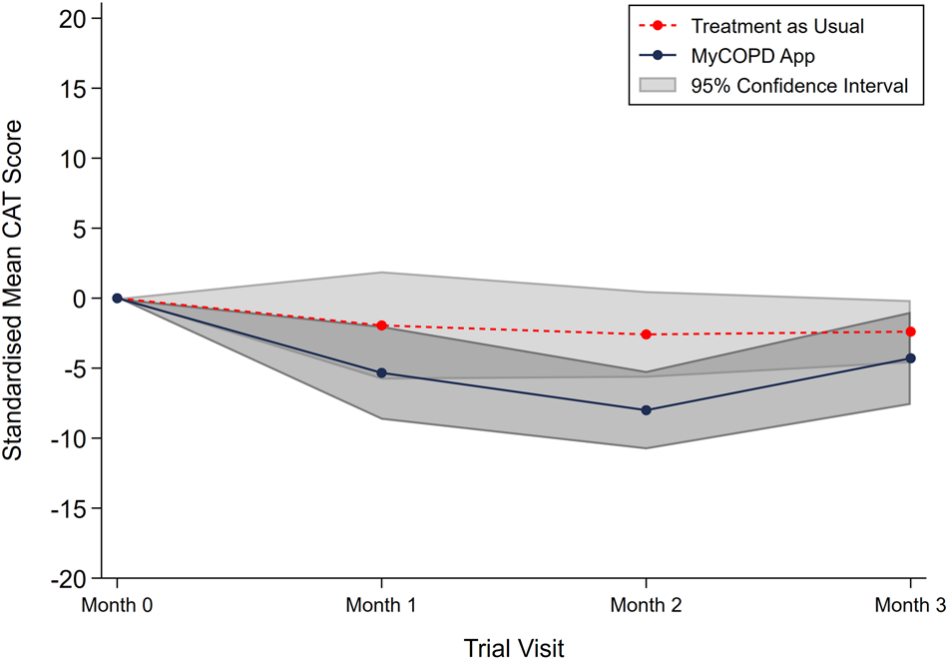
Standardised CAT score by trial arm over time. Indicated is the mean standardised CAT scores over the 3-month study in both the Treatment as Usual (Red) and MyCOPD App (Blue) arms. 95% confidence intervals are shown in light grey.

An assessment of the CAT score over the 90 day period, Fig. 3, found that the score was lower for the myCOPD arm at each study timepoint. The longitudinal analysis, which included all randomised participants and timepoints, showed the mean treatment difference for CAT score was 4.49 (95% CI: −8.41, −0.58, *n* = 41) lower in the myCOPD arm compared to TAU, indicating better disease control. When estimating the mean difference at the 90-day endpoint, which included 35 participants, the CAT score difference between arms was numerically lower at 2.94, Table 5, but the 95% CI included the value of no difference (95% CI: −6.92, 1.05, *n* = 35).

**Table 5:** Effectiveness outcome at 90 days by arm and adjusted mean difference.

Effectiveness Outcomes, 90 days mean (SD)	Treatment Group				3 month adjusted between arm difference	95% confidence interval
	TAU (*n* = 21)		MyCOPD (*n* = 20)			
CAT Score	25.1	(7.24)	20.7	(7.35)	−2.94	(−6.92, 1.04)
mMRC Score	2.78	(1.11)	2.76	(1.35)	0.0183	(−0.759, 0.796)
PAM Score	56.1	(18.49)	64.7	(13.46)	5.02	(−8.28, 18.3)
HAD Score	18.1	(7.78)	15.5	(8.88)	−3.08	(−7.61, 1.45)
SGRQ Score	64.1	(15.94)	61.9	(14.93)	−1.48	(−7.82, 4.86)
WPAI	6.50	(2.98)	6.24	(2.68)	−0.496	(−2.21, 1.22)
VSAQ Score	2.95	(2.43)	2.94	(1.54)	−0.163	(−1.40, 1.07)
Readmission rate	0.39	(0.50)	0.24	(0.44)	0.383^‡^	(0.0738, 1.99)
No. of exacerbations	1.88	(1.84)	1.06	(0.83)	0.581^†^	(0.315, 1.07)
No. of critical errors	4.00	(4.97)	1.17	(1.70)	0.377^†^	(0.179, 1.04)

Data have been rounded to three significant figures.

Estimates are the mean difference at 90 days from an ANCOVA model adjusted for baseline score and stratification variables (COPD severity and smoking status).

^‡^Denotes estimate is an Odds Ratio, ^†^Denotes estimate is a Rate Ratio.

For mean differences, estimates less than 0 favour myCOPD, for Odds Ratio or Rate Ratio estimates less than 1 favour myCOPD

*CAT* COPD assessment test, *mMRC* modified MRC test for dyspnoea, *PAM* patient activated measures, *HAD* hospital anxiety and depression scale, *SGRQ* St Georges respiratory questionnaire, *WPAI* work productivity activity impairment, *VSAQ* veterans specific activity questionnaire.

There were 35 participants who reported a two-point improvement in the CAT score at any timepoint in the study after baseline, 18 (90%) and 17 (81%) in the myCOPD and TAU arm, respectively. Studies have evidenced that the minimal clinically important difference (MCID 2) of CAT score is a decrease of two points following pulmonary rehabilitation and recovery from an acute exacerbation of COPD^26^.

### Exacerbations

**Fig. 4:**
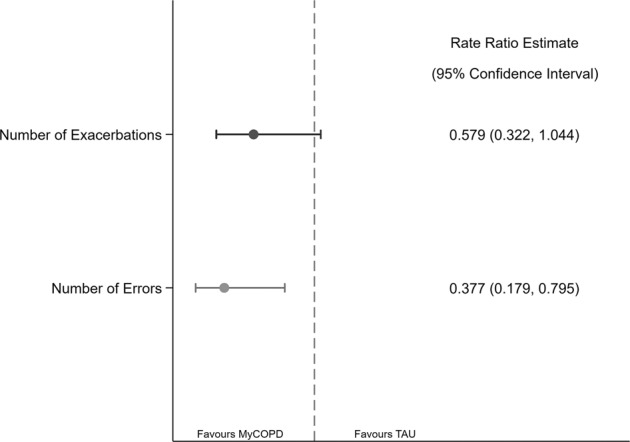
Rate ratio estimates of numbers of exacerbations and inhaler errors. Indicated are the incidence rate ratio estimates (and 95% confidence intervals) for number of exacerbations and inhaler errors.

Fewer exacerbations were observed post-intervention in both study arms, compared to baseline frequency, and the reduction was greater amongst participants in the myCOPD arm (Table 5). The adjusted Incidence Rate Ratio for exacerbations was 0.58 (95% CI: 0.32, 1.04, *n* = 35), see Fig. 4.

There were 4 (20%) readmissions of any length in the myCOPD arm and 7 (33%) in the TAU arm. The mean number of exacerbations was 1.86 and 1.08 for the TAU and myCOPD arms, respectively. The OR for readmission was 0.383 (95% CI: 0.074, 1.987, *n* = 35)

### Improved inhaler technique

The mean number of inhaler errors at 90 days was 4 and 1.2 for the TAU and myCOPD arm, respectively. The adjusted Incidence Rate Ratio, including an adjustment for number of inhalers and total number of errors, was 0.38 (95% CI: 0.18, 0.80, *n* = 35), see Fig. 4. The number of critical inhaler errors was greater according to the number of different inhalers used, which may mean participants duplicate errors across all their inhalers, inflating the effect of error ‘types’, although the mean number of inhalers was similar between arms at baseline and at 90 days.

### Other outcomes

There was an imbalance in the PAM score between arms at baseline, with higher scores in myCOPD arm than the TAU. The mean PAM score at 90 days was 64.7 and 56.1 in the myCOPD and TAU arms, respectively, and the adjusted mean difference at 90 days was 5.02 (95% CI: −8.28, 18.32; *n* = 35). Other patient reported measures are summarised in Table 5.

## Discussion

This feasibility randomised controlled trial explores the effects of digital tools in improving outcomes in COPD. Recent evidence has demonstrated the possibility of significant opportunities for clinical innovation through digital transformation which may alleviate the burden on healthcare systems as well as encouraging patients in self-management^18,19,24,25^.

However, clinical evidence appears limited, requiring further research to address reliable outcomes of enhanced self-management especially in high-risk patient populations recently discharged from acute care. This study has shown it is possible to use a digital platform such as the myCOPD app to support patients. Despite experiencing a significant clinical event such as exacerbation, the majority of subjects randomised to the app engaged with the system to a degree which enabled detection of clinically important benefits associated with this intervention.

Use of the app in this vulnerable patient population can be associated with detectable improvements in disease control measured by the validated CAT, reductions in exacerbations and a signal of reduction in risk of readmission to hospital. Measurement of inhaler technique was also shown to have improved in the digital arm compared to usual care.

In this study over 900 subjects admitted to hospital were considered by the local clinical service, of which 124 were deemed potentially suitable based on study entry criteria. Interestingly, of the 124 potentially eligible subjects, 66 declined study inclusion but also declined to give a reason, a further 15 stated they did not have time. Whilst these patients may have declined the inclusion into an RCT rather than access to the app per se, it illustrates that some patients with COPD may not feel confident to use digital tools to manage their health. Explanations for this may include privacy and security concerns, clinician engagement and patient fear and anxiety behaviours, especially after an acute COPD exacerbation, as well as challenges around low technology efficacy particularly in older adults^27,28^. However, the literature in this area is limited and requires further research.

A key topic of debate on the use of digital health interventions also lies around factors driving engagement, as well as the accessibility and availability of the technologies to individuals^29^. This is particularly pertinent in the setting of this study in which patients had been acutely unwell, unlike the majority of published trials to date which included stable patients^19,23,24,29^. There has been a revolution in the use of mobile digital technologies in the past decade with current availability of internet access to over 90% to the general UK population^28^. However, this picture is not evenly distributed across patient age groups or social classes. This is particularly relevant in the case of COPD where disease prevalence and severity track closely with older age and lower socioeconomic status^30^. A number of implementation experiments are currently underway in the UK to ensure that suitable access, not only to the hardware but also the skills, is available^27^. A health economic model of this intervention is yet to be developed but a simple consideration of the costs of readmission versus those of providing a tablet and app would suggest the prescription of hardware when needed is both reasonable and highly cost effective.

An uncertainty in the use of apps as health care intervention lies in the lack of understanding of the requirements for minimum patterns of patient use and interaction with the app to gain clinical benefit. Clearly, the clinical context, disease state, nature of outcome and intervention all play into the complexity of this question, but there is limited published data available. This study demonstrated that whilst 85% of the intervention arm were actively using the app in the first week, this had declined to 40% by three months. Interestingly however, active users continued to access the app several times per week and of course we are unaware of the real use of the usual care paper self-management guide. Indeed, even with this pattern of usage and the relatively short study period, clinically significant benefits were obtained and it is possible that patients used the system whilst perceiving an ongoing benefit. Further longer term studies are required to explore the threshold of use for benefit, the longevity of use and effect and innovations in app and service design which will be required to ensure sustained benefits are seen. Optimal clinical improvements in CAT score in the app arm were seen at 3 months. However, we may be seeing a loss of recurrent use as patients return to normal baseline after this period with only symptomatic or more severe patients continuing. We will aim to explore these patterns further in larger future studies to understand optimal use patterns to affect sustained improvements.

The association of the myCOPD app intervention with improvement in a number of clinical parameters is welcome but unexpected in a trial of this size. Previous studies of a range of digital interventions have failed to demonstrate significant effects on readmission rates for AECOPD in particular^7,31^. The exact mechanism by which the intervention manifests this effect is uncertain but may be linked to a number of aspects of improved self-management and medication usage. Inhaler technique is uniformly poor in patients with COPD, even those under the support of specialist services^32^. This signal was apparent in both treatment arms of the study with a high rate of critical inhaler errors, meaning that many patients were not delivering adequate amounts of inhaled therapy into their lungs due to their technique. Considering this pattern was apparent after recent hospital admission, when all patients spent many days under close medical scrutiny, this highlights the need for new approaches to training and maintaining good inhaler technique. The improvement in inhaler technique seen with the app suggests that repeated access to inhaler technique videos may be an effective way of addressing this important deficiency, which undoubtedly affects disease control in conditions such as asthma and COPD. Traditionally, inhaler training is provided by healthcare practitioners during face-to-face consultations with routine follow-up assessments to observe inhaler technique. However, this is unlikely to be scalable or repeatable. Additionally, inhaler training and subsequent assessments are often subjective and are dependent on the individual practitioners own experience and training^32^.

Despite an improvement in the inhaler technique, it is unlikely that this is the only mechanism by which clinical improvements in CAT score and exacerbation frequency were seen not least because inhaled therapies have had a limited direct effect on these outcomes in direct clinical trials and have failed to demonstrate benefits on hospital readmission^33,34^. The impacts seen therefore may be driven by a broader effect, including changes in activity, fitness and nutritional status as a consequence of patients accessing in-app educational material on these topics. We have previously shown that the app can deliver a pulmonary rehabilitation exercise programme comparable in outcomes to conventional face-to-face classes^25^. Whilst this material was available to the trial subjects through the app, we were not technically able to track specific access to these content at the time. Clearly, future research will enlighten the community as to the key ingredients and the consequent behaviours driving this positive signal in outcomes and will enable large scale delivery.

This small interventional study was limited in power to demonstrate effects on all measured outcomes but paves the way for a larger appropriately powered multicentre study. Interestingly, although a clear signal was seen in disease control – CAT score and exacerbations, other measures the PROs SGRQ, the Hospital Anxiety and Depression score, and the MRC breathlessness score did not change significantly. The reasons for this are likely to be complex but some scores, including the MRC tend to vary little over time and are related to severity of underlying disease than current control. The patient activation measure PAM which relates to an individual’s readiness to self-manage^35^ showed some changes in the app arm and a larger scale study may be required to explore the effect on this measure and how changes relate to clinical benefit. Additional work on process evaluation and patient feedback are already underway in further trials.

This study was unable to capture all indices for app usage and which domains of the complex intervention were accessed and were beneficial. The development of new technology has now enabled this with this app platform and future studies will offer valuable insights in these areas. The population of patients involved in this study came from a single UK centre where English was the predominant spoken language and lower socioeconomic status was ubiquitous. Further research will be required to determine the potential value of such interventions in other cultural settings and less deprived areas.

This randomised controlled trial of the myCOPD app sought to explore the feasibility and potential benefit of using a digital health intervention in a vulnerable patient population recently discharged from hospital, this will inform subsequent trial design. Early signals of efficacy and improved health supporting skills offer promise that larger trials may pave the way to addressing an enormous unmet clinical need in COPD and for digital tools to join mandated imaging^36,37^ and evidence-based interventions such as influenza vaccination^38–40^ and home oxygen therapy^41^.

## Methods

### Study design and participants

The study was approved by the research ethics committee for Berkshire B of the UK Health Research Authority (15/SC/0216 and registered online at ClinicalTrials.gov as NCT02706600). All subjects provided written informed consent. For protocol see Supplementary Fig. 1.

The study was a parallel arm feasibility randomised controlled trial with blinded outcome assessment at 90 days follow-up. The trial compared an online self-management support app; myCOPD, to conventional care with additional written support. Patients who had either been admitted to a single NHS Acute Trust in the UK or had been managed by the local COPD Admission Avoidance Team in a home-based environment with an acute exacerbation of COPD (AECOPD) were approached to participate in the study. The 6-month recruitment period commenced in June 2015 and closed in December 2015, resulting in study completion in March 2016.

Eligible patients willing to take part were issued with a Patient Invitation Letter, Patient Information Sheet and Informed Consent Form. Eligibility criteria included a primary COPD diagnosis as defined by NICE guidelines^42^ and using an inhaled device, 45 years or older, a current or ex-smoker for over 10 years and the ability to access and use an internet enabled device. Patient’s with an allergy to saccharin were excluded due to it being contained within placebo inhalers used to validate the inhaler technique.

Patients using myCOPD were registered by the healthcare team and self-activated via an email link. Once accessed, a ‘how to use’ video provides information on app content and usage, thereafter they are able to access the tile platform and utilise all aspects of the app by clicking on each tile and inputting their data. Tiles within the app are colour coded with images to indicate their content, how often they should be accessed and each tile contains a ‘how to use video’ (Supplementary Fig. 2). On every app login patients are automatically required to enter their symptoms for the day and must complete the COPD Assessment Test (CAT) questionnaire every four weeks before they are able to gain access to the platform tiles. Additionally, they are required to input their COPD related medication to support the development of their self-management plan. These features can be viewed in Supplementary Fig. 2.

CAT scores were used to determine outcome measures in response to pulmonary rehabilitation, recent exacerbation recovery progress and lifestyle changes. The scores range at 0–10 (mild), 11–20 (moderate), 21–30 (severe) and 31–40 (very severe)^42^.

### Randomisation and masking

Potential participants were consented and assessed for eligibility. Eligible subjects were then randomised using permuted blocks via an online randomisation system in a ratio of 1:1. Randomisation was stratified by smoking status and disease severity (FEV1 % predicted), defined by the global initiative for obstructive lung disease (GOLD) classification of COPD severity^30^. To ensure the study team remained blinded as to which arm of the study each participant was randomised to, the team was divided into two teams. One team (unblinded) was responsible for executing the initial visit, randomisation and liaising with participants with any study queries throughout the study and to deal with any potential adverse events. A separate blinded team member executed the final study visit. Participants were reminded in advance of the final visit for outcome assessment not to mention to the study team which arm of the study they were randomised to in order to preserve the single blinding.

### Procedures

Study visits took place in the research centre at Portsmouth Hospital NHS Foundation Trust or in the patient’s home. After screening and inclusion, participants underwent initial assessments by the clinical study team.

Assessment of inhaler technique was conducted for each type of device being used by participants at the time of study entry, each device was allocated critical errors in its usage. This list was comprised with assistance from a pharmaceutical company’s recommendations for use of their devices. Each participant in the study had their technique assessed for each inhaler device they currently used. Inhaler technique assessment was evaluated by an unblinded and blinded assessor.

After initial assessment participants who were randomised to the online arm were issued with a unique user name and password and were given basic instructions on how to access and use the app. Participants were provided with app access for 90 days and advised to use it as often as possible, or at least weekly, to familiarise themselves with their self-management plan and to view the online education and inhaler technique videos. The self-management plan encourages patients to learn which symptoms are normal for them and what to do if they experience a deterioration according to the data they have uploaded.

After the initial assessment, those who were randomised into the written arm were issued with a booklet with written education information about COPD and managing COPD, along with a written self-management plan. This took approximately 10–15 min to explain to the participant.

All participants were contacted by telephone at 30, 60 and 90 days to record CAT score and collect adverse and serious adverse events.

### Outcomes

A key aim of this study was to investigate the feasibility of undertaking a trial to determine the impact on patient outcomes of an interactive app-based patient self-management plan in comparison to a written self-management plan in current use. We looked at the activation rates of the app, use rates and patterns over time. We also aimed to obtain information on the distributions of effectiveness outcomes and preliminary evidence for the effectiveness of the digital app platform.

The CAT score was measured at four timepoints; baseline, 30 days, 60 days and at study completion (90 days), with the primary effectiveness being determined at the end of study. Secondary effectiveness outcomes were measured at baseline and study completion, and included inhaler technique; the St Georges Respiratory Questionnaire (SGRQ) which consists of 50 items with 76 weighted responses. This has good discriminative and evaluative properties and is responsive to therapeutic trials. It was developed and validated in both asthma and COPD and designed to measure the health impairment of patients with respiratory disease^43^; Patient Activation Measurement Tool (PAM) used for measuring the level of patient engagement in their healthcare. It was designed to assess an individual’s knowledge, skill and confidence for self-management. PAM is a 13-item scale that asks people about their beliefs, knowledge and confidence for engaging in a wide range of health behaviours and then assigns an activation score based on their responses to the 13-item scale^44^; Hospital Anxiety and Depression Questionnaire score (HAD) which is a clinical scale developed in 1983 and is in common use in clinical and trial settings. It consists of 7 questions scored from 0–3 to create a score out of 21. It is easily administered and has been well validated for the assessment of patients with COPD^45^; Veteran Specific Activity Questionnaire (VSAQ) which is a validated self-administered questionnaire developed to estimate exercise capacity for the development of exercise prescription. The VSAQ consists of physical activities listed in progressive order according to their energy demand, estimated by metabolic equivalents (METs). One MET is equal to resting oxygen consumption 3.5 ml/kg/min. Therefore, numbers of METs express the energy cost of physical activities as a multiple of the resting metabolic rate^46^, and Work Productivity Activity Impairment (WPAI) Questionnaire which is a well validated instrument to measure impairments in work and activities. The 6 questions relate to work absenteeism (hours missed work), work presenteeism (impairment whilst working) and work productivity lost due to a health condition^47^. The number of COPD exacerbations and the number of readmissions to hospital for COPD related events during the 90 day study period was captured during monthly phone calls and at the end of study visit. Only treated exacerbations were captured. Every effort was made to obtain 90-day follow-up data for all participants including those that were withdrawn from the trial by offering a home visit if required to obtain this information.

### Statistical analysis

The analysis for the trial feasibility outcomes was descriptive. Summary statistics were calculated to assess recruitment and retention rate, frequency of access to the online system and use over time of the myCOPD platform. The proportion of missing data by timepoint was calculated for key study variables (Supplementary Table 1).

All intervention effectiveness outcome measures were summarised by arm, using means and standard deviations or median (range, IQR) for continuous outcomes (as appropriate), and frequencies and proportions for categorical outcomes. As this was a feasibility trial and hypothesis testing was not the focus of this study, estimated differences between arms were only presented with 95% confidence intervals (CI) to indicate the uncertainty of the estimate, without testing for statistical significance. Analysis was undertaken using the intention-to-treat principle i.e. participants analysed in the arm to which they were randomised, regardless of their subsequent use of the intervention.

While not powered to perform hypothesis tests for effectiveness outcomes we did undertake analyses to estimate the mean difference between arms. The CAT score was analysed using a linear mixed model with baseline CAT score, stratification variables, treatment, the four timepoints and a treatment by timepoint interaction as fixed effects and a random subject effect using a symmetrical covariance matrix. With exceptions for inhaler error and exacerbations, all other secondary outcomes were assessed using an analysis of covariance model which was used to obtain an estimate for the mean difference at the final study visit (month 3), adjusted for baseline score and stratification variables; smoking status and COPD severity. For inhaler errors and exacerbations, count outcomes, a negative binomial regression, was used as there was evidence of overdispersion.

The number of serious adverse events (SAE) were tabulated by arm. Events were recoded using terms of the clinical investigators choosing.

### Reporting summary

Further information on research design is available in the Nature Research Reporting Summary linked to this article.

## Supplementary information

Reporting Summary

Supplemental Information

## Data Availability

All data relevant to the study are included in the article or uploaded as supplementary information.

## Code availability

The source code can has previously been published and can also be accessed by the mymhealth website (https://mymhealth.com/mycopd).
