# Author Correction: A randomised controlled feasibility trial of E-health application supported care vs usual care after exacerbation of COPD: the RESCUE trial

**DOI:** 10.1038/s41746-020-00360-w

**Published:** 2020-11-12

**Authors:** Mal North, Simon Bourne, Ben Green, Anoop J. Chauhan, Tom Brown, Jonathan Winter, Tom Jones, Dan Neville, Alison Blythin, Alastair Watson, Matthew Johnson, David Culliford, Jack Elkes, Victoria Cornelius, Tom M. A. Wilkinson

**Affiliations:** 1 my mhealth Limited, Bournemouth, UK; 2 Portsmouth Hospitals NHS Trust, Portsmouth, UK; 3 NIHR ARC Wessex, University of Southampton, Southampton, UK; 4 University of Southampton Faculty of Medicine, Southampton, UK; 5 Imperial College London, London, UK

**Keywords:** Respiratory tract diseases, Disease prevention

Correction to: *npj Digital Medicine* 10.1038/s41746-020-00347-7, published online 30 October 2020

The original version of the published Article had an error in the Abstract. The tenth sentence of the Abstract has been corrected to the following: Exacerbations tended to be less frequent in the digital arm compared to usual care; 18 vs 34 events. The HTML and PDF versions of the Article have been corrected.

