# Supplementary material for: A randomised controlled feasibility trial of E-health application supported care vs usual care after exacerbation of COPD: the RESCUE trial
Source: NPJ Digit Med. 2020 Oct 30;3:145. doi: 10.1038/s41746-020-00347-7 (PMC7603326; doi:10.1038/s41746-020-00347-7)
Supplement: Supplementary file 1 — Reporting Summary [file 41746_2020_347_MOESM1_ESM.pdf]

nature research

Corresponding author(s): Tom Wilkinson

Last updated by author(s): May 9, 2020

Reporting Summary

Nature Research wishes to improve the reproducibility of the work that we publish. This form provides structure for consistency and transparency in reporting. For further information on Nature Research policies, see our Editorial Policies and the Editorial Policy Checklist.

Statistics

For all statistical analyses, confirm that the following items are present in the figure legend, table legend, main text, or Methods section.

n/a	Confirmed	
☐	☒	The exact sample size (*n*) for each experimental group/condition, given as a discrete number and unit of measurement
☒	☐	A statement on whether measurements were taken from distinct samples or whether the same sample was measured repeatedly
☐	☒	The statistical test(s) used AND whether they are one- or two-sided<br>*Only common tests should be described solely by name; describe more complex techniques in the Methods section.*
☐	☒	A description of all covariates tested
☒	☐	A description of any assumptions or corrections, such as tests of normality and adjustment for multiple comparisons
☐	☒	A full description of the statistical parameters including central tendency (e.g. means) or other basic estimates (e.g. regression coefficient) AND variation (e.g. standard deviation) or associated estimates of uncertainty (e.g. confidence intervals)
☐	☒	For null hypothesis testing, the test statistic (e.g. F, t, r) with confidence intervals, effect sizes, degrees of freedom and P value noted<br>*Give P values as exact values whenever suitable.*
☒	☐	For Bayesian analysis, information on the choice of priors and Markov chain Monte Carlo settings
☒	☐	For hierarchical and complex designs, identification of the appropriate level for tests and full reporting of outcomes
☒	☐	Estimates of effect sizes (e.g. Cohen's d, Pearson's r), indicating how they were calculated

Our web collection on statistics for biologists contains articles on many of the points above.

Software and code

Policy information about availability of computer code

Data collection	The source code can has previously been published (Bourne: 2017) and can also be accessed by the mymhealth website (https://mymhealth.com/mycopd)<br><br>Bourne S, DeVos R, North M, et al. Online versus face-to-face pulmonary rehabilitation for patients with chronic obstructive pulmonary disease: randomised controlled trial. BMJ Open 2017;7:e014580. doi: 10.1136/bmjopen-2016-014580
Data analysis	The source code can has previously been published (Bourne: 2017) and can also be accessed by the mymhealth website (https://mymhealth.com/mycopd)<br><br>Bourne S, DeVos R, North M, et al. Online versus face-to-face pulmonary rehabilitation for patients with chronic obstructive pulmonary disease: randomised controlled trial. BMJ Open 2017;7:e014580. doi: 10.1136/bmjopen-2016-014580

For manuscripts utilizing custom algorithms or software that are central to the research but not yet described in published literature, software must be made available to editors and reviewers. We strongly encourage code deposition in a community repository (e.g. GitHub). See the Nature Research guidelines for submitting code & software for further information.

Data

Policy information about availability of data

All manuscripts must include a data availability statement. This statement should provide the following information, where applicable:

- Accession codes, unique identifiers, or web links for publicly available datasets
- A list of figures that have associated raw data
- A description of any restrictions on data availability

All data relevant to the study are included in the article or uploaded as supplementary information.

Field-specific reporting

Please select the one below that is the best fit for your research. If you are not sure, read the appropriate sections before making your selection.

☒ Life sciences ☐ Behavioural & social sciences ☐ Ecological, evolutionary & environmental sciences

For a reference copy of the document with all sections, see nature.com/documents/nr-reporting-summary-flat.pdf

Life sciences study design

All studies must disclose on these points even when the disclosure is negative.

Sample size	This was a faesibility trial which contained 41 participants, 21 were randomised to Treatment-As-Usual (TAU) and 20 to the myCOPD app
Data exclusions	No data was excluded from the analysis
Replication	This was a randomized controlled trial and data was captured from human participants using an app
Randomization	Potential participants were consented and assessed for eligibility. Eligible subjects were then randomised using permuted blocks via an online randomisation system in a ratio of 1:1. Randomisation was stratified by smoking status and disease severity (FEV1 % predicted), defined by the global initiative for obstructive lung disease (GOLD) classification of COPD severity
Blinding	. To ensure the study team remained blinded as to which arm of the study each participant was randomised to, the team was divided into two teams. One team (unblinded) was responsible for executing the initial visit, randomisation and liaising with participants with any study queries throughout the study and to deal with any potential adverse events. A separate blinded team member executed the final study visit. Participants were reminded in advance of the final visit for outcome assessment not to mention to the study team which arm of the study they were randomised to in order to preserve the single blinding.

Reporting for specific materials, systems and methods

We require information from authors about some types of materials, experimental systems and methods used in many studies. Here, indicate whether each material, system or method listed is relevant to your study. If you are not sure if a list item applies to your research, read the appropriate section before selecting a response.

Materials & experimental systems

n/a	Involved in the study	
☒	☐	Antibodies
☒	☐	Eukaryotic cell lines
☒	☐	Palaeontology and archaeology
☒	☐	Animals and other organisms
☐	☒	Human research participants
☐	☒	Clinical data
☒	☐	Dual use research of concern

Methods

n/a	Involved in the study	
☒	☐	ChIP-seq
☒	☐	Flow cytometry
☒	☐	MRI-based neuroimaging

Human research participants

Policy information about studies involving human research participants

Population characteristics	Age, years<br>We have reported the following baseline characteristics:<br>Sex<br>Female<br>Male<br>COPD Severity

	Moderate<br>Severe<br>Very Severe<br>Smoking Status<br>Current<br>Ex<br>Pack Years<br>FEV1<br>FEV1 % Predicted<br>FVC<br>FVC % Predicted<br>COPD Assessment Test (CAT) Score<br>Patient Activation Measure (PAM)<br>Modified MRC scale for Dyspnoea<br>St Georges Respiratory Questionnaire (SGRQ)<br>Hospital Anxiety and Depression scale (HAD)<br>Work Productivity Activity Impairment Questionnaire (WPAI)<br>Veterans Specific Activity Questionnaire (VSAQ) METs<br>Veterans Specific Activity Questionnaire (VSAQ) Score<br>Number of recorded Exacerbations<br>Total Number of Errors
Recruitment	Patients who had either been admitted to a single NHS Acute Trust in the UK or had been managed by the local COPD Admission Avoidance Team in a home-based environment with an acute exacerbation of COPD (AECOPD) were approached to participate in the study. The six month recruitment period commenced in June 2015 and closed in December 2015, resulting in study completion in March 2016.
Ethics oversight	The study was approved by the research ethics committee for Berkshire B of the UK Health Research Authority (15/SC/0216 and registered online at ClinicalTrials.gov as NCT02706600

Note that full information on the approval of the study protocol must also be provided in the manuscript.

Clinical data

Policy information about clinical studies

All manuscripts should comply with the ICMJE guidelines for publication of clinical research and a completed CONSORT checklist must be included with all submissions.

Clinical trial registration	NCT02706600
Study protocol	The clinical trial is registered online at ClinicalTrials.gov as NCT02706600
Data collection	The six month recruitment period commenced in June 2015 and closed in December 2015, resulting in study completion in March 2016. Data from recruited patients was recorded from baseline to a 90 day period
Outcomes	The primary predefined outcomes involved feasibility measures, thus we looked at the activation rates of the app, use rates and patterns over time. For secondary outocomes we looked at predefined outcomes on the distributions of effectiveness outcomes and preliminary evidence for the effectiveness of the digital app platform including CAT scores.
