# Supplementary material for: A randomised controlled feasibility trial of E-health application supported care vs usual care after exacerbation of COPD: the RESCUE trial
Source: NPJ Digit Med. 2020 Oct 30;3:145. doi: 10.1038/s41746-020-00347-7 (PMC7603326; doi:10.1038/s41746-020-00347-7)
Supplement: Supplementary file 2 — Supplemental Information [file 41746_2020_347_MOESM2_ESM.pdf]

Trial Protocol

RESCUE-COPD 1

A Randomised controlled Feasibility trial of E-health platform Supported Care vs Usual care after Exacerbation of **COPD**

Chief Investigator:	Professor Anoop Chauhan
Principle Investigator:	Dr Ben Green
Sponsor:	My mhealth Limited
Co-Investigators:	Dr Tom Wilkinson<br>Dr Simon Bourne<br>Dr Thomas Brown<br>Mal North RN
Study Statistician:	Dr Victoria Cornelius

Protocol Version:	2
Protocol date:	11/FEB/2105
Minor Amendment date	23/APRIL/2015
Study Number:	my mhealth-RESCUE COPD -001
Issue Date:	11/FEB/2015

Study Synopsis

Study Code Number:	RESCUE COPD 001
REC Number	15/SC/0216
UKCRN Number	PHT/2015/39
Title of the Trial	A randomised controlled feasibility trial of E-health platform Supported Care vs Usual care after Exacerbation of COPD
Development Phase:	Exploratory Clinical
Principle Investigator:	Dr Ben Green
Sponsor:	Portsmouth Hospitals NHS FT
Purpose of Clinical Trial	To assess the feasibility of undertaking a randomised controlled trial to determine the effectiveness of E-health platform Supported Care in improving patients outcomes after exacerbation of COPD
Objectives	Usability of platform<br>Symptom Control<br>Inhaler technique<br>Treatment adherence<br>Quality of Life
Trial Design:	A two parallel arms non-blinded randomised controlled feasibility trial
Planned Trial Period:	Participant follow up: 90 days approximately<br>Recruitment Period 6 months
Intervention (description, frequency, details of delivery)	The study will deliver two interventions one to each arm of the study. One group will be randomised to myCOPD an online self management system and the other group to a conventional written self management plan. Details of the interventions can be found on pages 14 and 15 and Appendix 3 of this protocol.
Outcomes:	Trial feasibility outcomes:<br>• Recruitment rate<br>• Withdrawal and loss to follow-up rate<br>• Usability of the of E-health platform

- Use of the of E-health platform
- Completeness of data collection

Intervention effectiveness outcomes

- COPD Assessment Tool (CAT) Score
- Improvement in number of critical errors for Inhaler Technique
- Quality of Life score: St Georges Respiratory Questionnaire (SGRQ)
- modified Medical Research Council Dyspnoea Questionnaire (mMRC)
- Patient Activation Measure (PAMs)
- Hospital Anxiety and Depression Score (HADS)
- COPD treatment adherence
- Safety

Sample size	60
Study Population:	• Adult, male and female COPD patients following admission to hospital or supported care with exacerbation<br>• Age Range 45-80 years
Study Procedures	• Informed Consent<br>• Clinical History and Physical Examination<br>• Inhaler Technique Assessment<br>• PROMs<br>• CAT<br>• SGRQ<br>• mMRC<br>• HAD<br>• PAM<br>• VSAQ<br>• WPAI<br>• SMP training<br>• Safety- AE & SAE capture<br>• Qualitative interview
Inclusion Criteria:	1. Subjects able to complete all study procedures and give written informed consent.<br>2. A diagnosis of COPD defined as per the NICE COPD guidelines<br>3. Male or female volunteers aged 45+ admitted to hospital or the ambulatory care service with a primary diagnosis of acute exacerbation of COPD<br>4. Patients who are taking inhaled therapies

	for COPD<br>5. Current or ex-smokers with a pack year history of more than 10 years<br>6. Access to the internet and ability to operate web platform and or use written action plan
Exclusion Criteria:	1. Patients who have any another respiratory disease as their main complaint such as asthma, bronchiectasis, lung cancer, tuberculosis or any other significant respiratory disease.<br>2. Patients unable to read or use an Internet enabled device.<br>3. Patients with any uncontrolled medical condition which in the view of the principle investigator or their team would confound the impact of a COPD directed support tool.<br>4. Sensitivity or allergy to saccharin
Study Schedule Outline:	Day 1: A detailed study schedule appears in **Appendix 1**.
Follow Up Duration:	90 days (+/- 7 days) after hospital discharge for COPD exacerbation
Statistical Methods:	Demography: descriptive statistics will be provided.<br>Adverse events: will be described descriptively.<br>ITT analysis of change of CAT score between treatment groups<br>ITT Analysis of incidence of critical errors in inhaler technique at d90<br>Comparative analysis of secondary and exploratory outcomes between study arms

Appendices:

One: Study Schedule

Two: PROMS

- St Georges Respiratory Questionnaire (SGRQ)
- COPD Assessment Tool (CAT)
- Modified MRC Questionnaire
- Hospital Anxiety and Depression Questionnaire

- Patient Activation Measure (PAMS)
- Work Productivity Activity Impairment Questionnaire (WPAI)
- Veterans Specific Activity Questionnaire

Three: Interventions

- MyCOPD solution screen shots
- COPD action plan- paper version

Four: Inhalers Critical Errors Lists

- Meter Dose Inhaler
- Meter Dose Inhaler plus Spacer
- Turbuhaler
- Handihaler
- Ellipta
- Respimat
- Nexthaler
- Easyhaler
- Easibreathe
- Autohaler
- Breezehaler
- Spiromax
- Genuair
- Accuhaler

List of Abbreviations

E – Health	Electronic Health
mHealth	Mobile Health
AE	Adverse Event
CAT	COPD Assessment Tool
COPD	Chronic Obstructive Pulmonary Disease
CRF	Case Report Form
CTM	Clinical Trial Material
EC	Ethics Committee
GCP	Good Clinical Practice
HAD	Hospital Anxiety and Depression score
ICH	International Conference on Harmonization
ID	Identification
IEC	Independent Ethics Committee
ITT	Intent To Treat
mMRC	Modified Medical Research Council Dyspnoea score
PAM	Patient Activity Measurement
PIC	Participant Identification Centre
PROMS	Patient Recorded Outcome Measurements
SAE	Serious Adverse Event
SAP	Statistical Analysis Plan
SGRQ	St Georges Respiratory Questionnaire
SMP	Self Management Plan
VSAQ	Veterans Specific Activity Questionnaire
WPAI	Work Productivity Activity Impairment Questionnaire

Introduction and Background

Aims and Objectives

The primary aim of this study is to investigate feasibility of undertaking a trial to determine the impact on patient outcomes of an interactive web-based patient self-management and action plan in comparison to a written action plan in current use. The usability of the platform and its effect on symptom control, inhaler technique, treatment adherence and quality of life will be studied in subjects recruited from patients recently admitted to hospital or to specialist ambulatory care for the treatment of an exacerbation of COPD, using validated tools and questionnaires. The safety and efficacy of the novel intervention will be reported and findings will contribute to the design and implementation of larger future studies.

Background and Significance

COPD is characterised by airflow obstruction that is not fully reversible. The airflow obstruction is usually progressive in the long term and results in the gradual development of limiting symptoms such as cough and breathlessness. Cigarette smoking is the predominant cause of COPD, but other factors, particularly occupational exposures, may also contribute to the development of COPD[1]. Exacerbations occur when there is a rapid and sustained worsening of symptoms beyond normal day-to-day variations, these are often associated with acute respiratory infections and can result in unscheduled health care use and hospitalisation.

There are currently 900,000 patients diagnosed with COPD in the UK.[2] COPD is the second leading cause for hospital admission in the UK; recent figures show that one in three people with COPD is readmitted within 28 days of discharge.[3] The latest Quality Standards for COPD from the National Institute for Health and Care Excellence (NICE) highlights the importance of self-management for patients and recommends that every person diagnosed with COPD be given an individualised comprehensive self-management plan.[4] Self-management has been shown to be associated with health service utilisation by COPD patients, improve their quality of life[5] and reduce hospitalisation.[6]

Patients with COPD are often prescribed a complex combination of inhaled medications in a variety of devices; these medications differ enormously in their delivery method and periodicity. This complexity contributes to poor compliance and therefor wasteful prescribing and it is estimated that there is currently only at best a 30% compliance to COPD medications.[7]

The majority of COPD action plans are delivered in a written (paper) format, and currently a validated online interactive solution does not exist. *MyCOPD* has been designed and programmed by my mhealth Ltd with patient and clinician involvement. It is a web-based self-management system designed to encompass the principles of supporting self-care in people with COPD. The system can be easily and securely accessed online by patients and healthcare

professionals to facilitate the effective recognition of symptoms, inhaler technique and management of medicine for patients with COPD. In 2013 myCOPD was tested in a COPD population as part of a service development project. 36 people with COPD tested myCOPD and the results of the study showed 95% demonstrated a decrease in symptom score and an overall improvement in inhaler technique.[8] There are numerous written self management plans available which have been developed and produced by individual Trusts, GP surgeries and pharmaceutical companies. The written Self management plan we intend to use for comparison was developed by HealthQuest Solutions Ltd in partnership with Novartis, as part of a national programme. This self management plan to date has been delivered to over 50,000 patients across the UK.

An initial design and optimisation phase of MyCOPD development demonstrated that the tool is usable in this patient group. Patients testing the platform demonstrated regular and effective daily use with associated improvements in inhaler technique and symptom control. Patients fed back that the platform was easy to use, reliable and that it would become part of their daily treatment regime if available.

Purpose of trial

In a group of patients with COPD, discharged from hospital with an acute exacerbation at their 2 week review we will undertake a feasibility trial to test the design and obtain preliminary evidence that the MyCOPD web based self-management plan will result in greater improvements in quality of life scores, faster recovery to baseline, and fewer errors in inhaler technique compared to conventional paper based plans. The result from this trail will inform the design and sample size calculation for a definitive trial to determine the effectiveness MyCOPD

<u>Study Objectives and Outcomes</u>

<u>Objectives</u>

The overarching objective of this trial is to assess the feasibility of undertaking a randomised controlled trial of an E-health platform Supported Care to improve patient outcomes after discharge from hospital or post exacerbation.

The specific objectives are:

- To estimate the recruitment and retention rate
- To assess the usability and use of the E-health platform
- To assess completeness of the study data collected
- To obtain information on the distributions of effectiveness outcomes and preliminary evidence for the effectiveness of the E-health platform
- To determine the most suitable primary outcome for the definitive trial

Endpoints:

As this is a feasibility trial no primary outcome is specified and we will use this trial as an opportunity to select a suitable primary outcome for the definitive trial.

Feasibility endpoints:
- Recruitment rate
- Withdrawal and lost to follow-up rate by study completion (90 days from discharge (+/- 7))
- Usability of the E-health platform assessed using patient feedback
- The frequency and duration that the E-health platform was accessed by patients until study completion (90 days from discharge (+/- 7))
- Proportion of missing data by variable and time point.

Intervention effectiveness outcomes
- The time to a two-point improvement in the COPD Assessment Tool (CAT) Score
- The improvement in CAT score at study completion 90(+/- 7) days in the MyCOPD support population compared to usual care
- The number of critical errors by inhaler at study completion 90(+/- 7)
- St Georges Respiratory Questionnaire (SGRQ) score at study completion 90(+/- 7)
- Modified MRC dyspnoea score at study completion 90(+/- 7)
- Patient Activation Measure (PAMs) score at study completion 90(+/- 7)
- Hospital Anxiety and Depression Score (HADS) at study completion 90(+/- 7)
- WPAI score at study completion 90 (+/- 7)
- VSAQ score at study completion 90 (+/- 7)
- COPD treatment adherence (what would be monitored and how would this be measured?)
- The number of exacerbations by study completion 90(+/- 7) days
- The time to exacerbations
- The rate of and timing of antibiotic use by study completion 90(+/- 7) days
- The rate of and timing of steroid use by study completion 90(+/- 7) days

Safety the number of AEs and SAEs by study completion 90(+/- 7) days

Exploratory Objectives and Outcomes

The exploratory objectives of the study are to determine potential patient reported improvements in the web based and paper based support tools and to investigate the impact of each platform on decisions to seek unscheduled health care support.

- Exploratory Outcomes- qualitative capture of patient's experience of use of each support platform, suggested improvements and decision processes regarding accessing healthcare.

Study Population

Potentially suitable patients will be identified by the clinical respiratory team at Portsmouth Hospital during admission for the management of acute exacerbation. Patients will be given clinical trial information (PIS) prior to hospital discharge and those patients who have expressed an interest in study participation will be contacted by the trial team within two weeks of discharge to schedule the consent and screening visit.

Up to 60 patients will be recruited in total and therefore an excess of this number may need to be screened to ensure eligibility.

Inclusion Criteria

Inclusion of subjects into this clinical trial will be judged by and will be at the discretion of the Principal Investigator.

1. Subjects able to complete all study procedures and give written informed consent.
2. A diagnosis of COPD defined as per the NICE COPD guidelines
3. Male or female volunteers aged 45+ admitted to hospital or the ambulatory care service with a primary diagnosis of acute exacerbation of COPD
4. Patients who are taking inhaled therapies for COPD
5. Current or ex-smokers with a pack year history of more than 10 years
6. Access to the internet and ability to operate web platform and or use written action plan

Exclusion Criteria

1. Patients who have any another respiratory disease as their main complaint such as asthma, bronchiectasis, lung cancer, tuberculosis or any other significant respiratory disease.
2. Patients unable to read or use an Internet enabled device.
3. Patients with any uncontrolled medical condition which in the view of the principle investigator or their team would confound the impact of a COPD directed support tool.
4. Patients who are currently recruited to other research studies.
5. Patients who have finished a study within the last three months.
6. Patients who have a sensitivity or allergy to saccharin.

Definition of Acute Exacerbation of COPD

The identification of potential study participants will be conducted by the clinical respiratory team and respiratory research team at Portsmouth Hospitals trust under the approval of the respiratory consultants. Patients with known, spirometrically confirmed COPD with a primary diagnosis of acute exacerbation will be approached by the team. The diagnostic criteria for acute exacerbation are therefore clinical and the PI will determine the suitability for inclusion.
During the study patients may experience recurrent or further exacerbations, the diagnosis and management of these will be under the direction of the usual clinical services (primary and secondary) these will be captured by the study team.

Withdrawal of subjects from the study

Participation in this study may be discontinued for any of the following reasons:

- Intolerable adverse events due to the study procedures(AEs).
- The wish of the subject.
- Non-compliance with study procedures.
- Investigator' decision that withdrawal from further participation would be in the subject's best interest.
- Development of inter-current illness, which in the opinion of the Investigator would confound the study outcome data.
- Termination of the study by the Investigator or Sponsor.

A subject can withdraw from the study at any time, for any reason, without prejudice to their future medical care.

Those who withdraw will be required to return if allocated their tablet computers.

Study Design and Conduct

Recruitment and Informed Consent

Day 1 Case Identification:

Patients admitted to the respiratory clinical service at Portsmouth Hospital trust with a pre-existing diagnosis of COPD and with a primary or co-primary diagnosis of acute exacerbation will be identified by the clinical and respiratory research teams in the Trust. Potentially eligible patients will be provided with written information about the study in the form of the patient information sheet. The patients contact details will be recorded and patients contacted within two weeks of discharge by telephone and/or written communication and/or email. Participants who are not admitted but treated for an exacerbation of their COPD in primary care will also be recruited from PIC sites at:

- Kirklands Practice (Portsmouth)
- Queenswood Practice (Cowplain)
- Stoke Road Practice (Gosport)
- Badgerswood Practice (Bordon)
- Forest Surgery (Bordon)

We will also present the study to local Breathe Easy Groups in Portsmouth and invite their members to participate in the study should they exacerbate. We will give out letters of invite to the group giving them the opportunity to take part in the study should they at any time during the recruitment phase exacerbate.

Patients wishing to participate in the study will be invited to attend the consent and screening.

Visit 1- Consent and Screening

Patients wishing to participate as above, will within 2 weeks of hospital discharge (+/- 1 week) at Visit 1 initially give written informed consent. This visit may occur at the hospital, in primary care or at the patient's home depending on patient preference.

Clinical data will be captured. Symptom scores will be calculated using the COPD Assessment Tool (CAT), SGRQ, modified Medical Research Council Dyspnoea Questionnaire (mMRC) Patient Activation Measure (PAM), Work Productivity Activity Impairment (WPAI) and the Veterans Specific Activity Questionnaire(VSAQ). At this visit the team will also record the admission and exacerbation history, video the inhaler technique and complete an inhaler critical errors assessment.

Randomisation

Patients will be randomised to myCOPD E-health platform or written self-support platform at the end of visit 1. Randomisation will take place using permuted blocks in a ratio of 1:1 to either online SMP (myCOPD) or written SMP (Standard of Care). A member of the study staff will randomise participants using an online randomisation system developed and hosted by my mhealth Ltd. Participants will be issued with a randomised identifier of the following format:
[A-Z][A-Z\-][A-Z]\.[0-9]{4}\.[A-Z]{1,10}
meaning 3 characters for subject initials followed by 4 randomised sequential digits,
Examples: A-T 0044 (online SMP) or DME 0017 (written SMP). These codes will be stored on the my mHealth secure server

Emergency code break will not be required in this study, due to the nature of the intervention.

Visit 1 – Treatment Allocation and Training

Patients will receive home visit formalised training on the use of the appropriate platform be loaned and be provided with online instruction for use. Initial understanding and competence will be assessed and recorded. The time taken to achieve competence will be recorded.

Visit 2 and 3 - Study and Telephone Support

All participants will be given the contact details of the clinical trial team. The team will be available for technical support and advice during office hours for the duration of the study. The clinical care of the patient will remain under the usual clinical service. The patients GP and supervisory consultant will be informed of the patient's participation in the study and any clinical advice requested by the patient during the study.

Patients will continue with usual medical care as prescribed and supervised by the usual clinical service. This may include the use of pre-prescribed rescue medication for exacerbations of COPD (oral corticoid-steroids and oral antibiotics) in a 'rescue pack'. Both written and electronic plans support the potential use of this treatment intervention as directed by the prescribing

physician.

If patients request face to face support for technical assistance an extra visit may be conducted on site or at the patient's home. All additional contact time and interaction will be recorded.

All patients will receive a monthly (every 30 days (+/- 3 days) call to record active study participation and address queries. AEs and SAEs, and CAT score will be recorded. Exacerbations and additional prescribed medications, unscheduled health care use, GP visits and ED attendances will be recorded.

Visit 4 – Study Completion

At 90 days after the initial study (enrolment) visit (+/- 7 days) the patient will attend the study site or be visited for the final assessment. This assessment will include the same measures as Visit 1.

- Record clinical data
- SAEs AEs and exacerbations
- Unscheduled health care use
- CAT score
- mMRC
- HAD
- PAM
- SGRQ
- WPAI
- VSAQ
- Inhaler technique assessment
- A practitioner blinded to the intervention arm will perform assessment of inhaler technique videos. They will use the critical error list to evaluate technique.

After PROMs are completed patients will be asked to feedback to the researcher on the platform used in the study, any suggestions for improvement in design and reflections on how the platform affected their self-care decisions during the study. These inputs will be written down as free text and recorded and/or videoed. The study team will then collect the loaned computer tablets from those participants randomised to the myCOPD arm and deliver conventional written self management plans to those participants.

Clinical Data and PROMs

Clinical data including history, treatments lung function assessments and co-morbidities will be recorded from the clinical notes, use of electronic records or GP records.

COPD Assessment Tool (CAT) Questionnaire

This is a validated symptom scoring system used in COPD studies[9] The CAT questionnaire contains eight questions and provides a reliable measure of the impact of COPD on health status. Patients read the two statements for each item, which describe the best and worst scenario, (e.g I never cough – I cough all the time), and decide where on the scale of 0-5 they fit. The maximum score is out of 40. The higher the CAT score the greater the impact of symptoms on their health status. Experts involved in the development of CAT suggest that any change of 2 or more in the patient's final score may indicate a clinically significant change.[9]

Modified Medical Research Council Dyspnoea Questionnaire (mMRC)

The mMRC dyspnoea scale comprises five statements that describe almost the entire range of respiratory disability from none (Grade 0) to almost complete incapacity (Grade 4). It can be self-administered by asking subjects to choose a phrase that best describes their degree of breathlessness associated with activity[10].

St Georges Respiratory Questionnaire (SGRQ)

The SGRQ questionnaire has 50 items with 76 weighted responses. It has good discriminative and evaluative properties and is responsive to therapeutic trials. It was developed and validated in both asthma and COPD and designed to measure the health impairment of patients with respiratory disease.[11]

Hospital Anxiety and Depression Scale (HAD)

This clinical scale was developed in 1983 and is in common use in clinical and trial settings. It consists of 7 questions scored form 0-3 to create a score out of 21. It is easily administered and has been well validated for the assessment of patients with COPD.[12]

The Patient Activation Measure (PAM)

PAM is a tool used for measuring the level of patient engagement in their healthcare. It was designed to assess an individual's knowledge, skill and confidence for self-management. PAM is a 13-item scale that asks people about their beliefs, knowledge and confidence for engaging in a wide range of health behaviors and then assigns an activation score based on their responses to the 13-item scale.[13]

Work Productivity Activity Impairment (WPAI)

The Work Productivity and Activity Impairment (WPAI) questionnaire is a well validated instrument to measure impairments in work and activities. The 6 questions relate to work absenteeism (hours missed work) Work presenteeism (impairment whilst working) and work productivity lost due to a health condition.[14]

Veterans Specific Activity Questionnaire (VSAQ)

The VSAQ is validated Self administered questionnaire developed to estimate exercise capacity for the development of exercise prescription. The VSAQ consists of physical activities listed in progressive order according to their

energy demand estimated by metabolic equivalents (METs). One MET is equal to resting oxygen consumption 3.5 ml/kg/min. Therefore, numbers of METs express the energy cost of physical activities as a multiple of the resting metabolic rate.[15]

Inhaler Technique

As different inhaled medication devices require different techniques, each participant's inhaler technique will be assessed according to manufactures guidelines for each device and critical errors recorded. Their technique will be video recorded at visit one and visit two. Any changes to devices during the study will be recorded prior to starting any new treatment. Each participant's technique will be evaluated as either GOOD or POOR and the number of critical errors recorded. Each device will have its own critical error list we have created based on manufactures guidelines.(Appendix 4)

Exacerbation History

The numbers of COPD exacerbations requiring oral antibiotics and/or oral steroids by the participants one year prior to starting the study will be recorded at visit one. If the participant has any exacerbations requiring oral antibiotics and/or steroids required during the period of the study this will be recorded at visit two.

Admission History

Admission and readmission data will be collected at visit 1 and visit 2 and verified by the GP patient records or other source data.

Assessing Inhaler Technique

Assessing inhaler technique can be subjective we have developed a critical error list for all inhaler devices licenced for COPD to assist study the study team in the evaluation of technique. Each device will have its own critical error list based on manufactures guidelines. The list can be found in appendix 4 of the study protocol.

Interventions: Patient Support Tools – Appendix Three

MyCOPD Solution- web based patient support tool

myCOPD is a system that can be accessed by patients using any device that can connect to the internet and can operate in any internet browser. It contains: Educational information on

- What is COPD
- Breathing Anatomy
- Your Lungs
- COPD symptoms
- Diagnosing COPD

- Exacerbations and treating exacerbations
- Breathlessness and Anxiety
- Sputum Clearance
- Staying Well with COPD
- Inhalers and treatments for COPD
- Keeping Active
- Pulmonary Rehabilitation

In addition to this there are:

Inhaler technique videos explaining the correct technique required to use different inhaler devices licensed for use in people with COPD.

Medication and symptom diaries.

Appointment diary

Bitesize pulmonary rehabilitation videos designed to promote and support exercises that can be done in a home setting.

Oxygen Alert Card – Users can create their own oxygen alert card online

A 5 Day local weather and pollution reports - Feed for reports come from the met office and DEFRA.

A Self management plan, which consists of a traffic light system to direct patients to the most appropriate action to take should their symptoms deteriorate. The action plan is populated automatically with the information input by the patients. (Appendix 3)

HealthQuest Written COPD Action Plan

The HealthQuest written self management plan was produced in 2013. It is a one page document which contains a written self management plan which can be individualised for the patient. It consists of a traffic light system to direct patients to the most appropriate action to take should their symptoms deteriorate.

Trial Centres and Sites

- Portsmouth Hospitals NHS FT and affiliated centres
- Primary Care Centres in Portsmouth
  - Kirklands Practice (portsmouth)
  - Queenswood Practice (Cowplain)
  - Stoke Road Practice (Gosport)
  - Badgerswood Practice (Bordon)
  - Forest Surgeries (Bordon)
- Patient's Homes

Data Management and Statistical Analysis

Record keeping, monitoring, and record retention

Appropriately qualified individuals will be utilised to supervise the overall conduct of the trial, to handle the data, to verify the data, to conduct the statistical analyses, and to prepare the trial reports.

MyCOPD- Web-based platform

myCOPD Security Controls
This section outlines the security controls in the operational lifetime of the myCOPD functionality for research.

1. **ISO-27001**
HealthQuest Solutions implements an Information Security Management System (ISMS) under the Data Protection Act 1998. Current policies include an Access Control Policy, a Data Governance Policy and a Password Policy.

2. **Secure Sockets Layer (SSL)-encrypted communications**
The functionality uses a business-level SSL digital certificate to ensure that all communications are protected. This is enforced both by the system that receives the requests and software that handles the request.

3. **Access model**
The system implements an RBAC (Role Based Access Control) access model. Data requests are checked against user and role permissions to view, insert, update or delete data. The system architecture provides permission checking by denying access to all data, and then explicitly granting access to specific operations for specific users.

4. **Prevention of XSS (Cross-Site Scripting) attacks, viruses, and session theft**
The system provides centralized bidirectional escaping of all text. This means that a potential attacker is unable to embed code. This control is applied to all the text, either resultant of a database query or submitted by a user.
Every uploaded file and every file served is previously analysed at the file content header level. This ensures that executable files injected through a system break-in or an upload won't have any effect.
There are specific controls in place to prevent session forgery, web sessions are represented as salted hash of key/values. The system uses different secret salts for development and deployment. Cookies aren't used for data that expose patient personal information or log-in credentials.

5. **Password storing**
The system does not store passwords in plain text. Therefore password recovery is not available. If the users forgets or loses their password account recovery will require the generation of a new password.

6. **Backup security**
Local and remote backups of patient data are stored in encrypted file systems that require passwords for decryption. Back ups are performed daily

Confidentiality
All data collected during the study will be coded with unique study numbers to ensure complete confidentiality of study subjects. The confidentiality of the subject will be respected and maintained at all times.

All required subject data will be recorded on case report forms (CRFs) or other study-specific media. Data will be retained for the ICH specified period.

Statistical Section [16,17,18,19]

Randomisation

Patients will be randomised to MyCOPD E-health platform or written self-support platform at the end of visit 1. Randomisation will take place using permuted blocks in a ratio of 1:1 to either platform MyCOPD or written plan (Standard of Care).

Once a patient is consented, all baseline data collected and eligibility confirmed, a study staff will randomise participants to the study using an online randomisation system developed and hosted by my mhealth Ltd.

As this is a web based tool the study staff member will log in to the website using their unique user code and password. They will then entre the participants screening number, initials and the date of the visit. This will then generate a unique randomisation number and inform the user, which study arm the participant will receive either written SMP or online SMP.

Emergency code break will not be required in this study, due to the nature of the intervention.

Sample size rationale

In this trial the main aim is to assess the feasibility of undertaking a fully powered trial in this area. As a result the sample size has been chosen with consideration to estimating a number of parameters with sufficient precision and not based on the number required to test a hypothesis.

If we approach one hundred and twenty participants to take part in this trial and assume a recruitment rate of 50% this would enable us to estimate the 95% confidence intervals for the recruitment rate with precision of at least ± 9 percentage points. With 60 participants recruited to the trial (n=30 per arm), we would be able to estimate the 95% confidence intervals for the withdrawal/dropout rate with precision of at least ± 7 percentage points if the true withdrawal/dropout rate was 9%.

We also would like to obtain a sample size sufficient to obtain a good estimate for the variance of each of the potential primary outcomes. The variance estimate of the chosen primary outcome would then be used to inform the power calculation for a definitive trial. Guidelines for sample size to estimate

the variance vary, but typically range between 24-50 participants as a result we think that 30 participants per group will be sufficient.[12,13,14,15]

We will also estimate the difference between arms with 95% confidence intervals. In our pilot work we observed that CAT had a standard deviation of 6.5, if this is observation is found to hold in this study we would be able to estimate the difference in CAT between arms with 95% confidence interval width of +/- 3.3.

Statistical Methods

The analysis of the trial feasibility aims will be primarily descriptive. Summary statistics will be calculated to assess aims such as the recruitment and retention rate, frequency of access and use over time of the E-health platform. A list of key study variables will be drawn up and the proportion missing by time point and arm will be calculated.

All intervention effectiveness outcome measures will be summarised by intervention arm using means and standard deviations or median (range, IQR) for continuous outcomes (as appropriate), and frequencies and proportions for categorical outcomes. As this is a feasibility study with only 30 participants per arm the trial will be underpowered to detect statistically significant differences in outcome measures between arms, however the difference between arms will estimated and presented with 95% confidence intervals to indicate the uncertainty of the estimate. The estimation analyses will be undertaken using an intention-to-treat approach i.e. participants will be analysed in the arm to which they were randomised regardless of whether or not they subsequently used self-help online or via usual care approach or their adherence to the intervention.

The time to a two point increase in CAT score will be will be plotted using the method of Kaplan-Meier and Cox proportional hazards model will be used to estimate the relative 'risk' between arms by means of a hazard ratio. An analysis of covariance model will be used to obtain an estimate for the mean difference in CAT score at final study visit (90 days) between the two intervention arms adjusted for baseline CAT score. In addition CAT values over time will be plotted for individual and the mean value over time displayed by intervention arm. A longitudinal analysis will then be undertaken using a linear mixed model including baseline CAT as a covariate with random subject effects using (if possible) a structured (symmetrical) covariance matrix. Model assumptions will be assessed thorough residual analysis.

Every effort will be made to obtain 90- day follow up data for all participants including those that have withdrawn from the trial by offering a home visit if required to obtain this information.

The mean difference in the number of critical errors between arms will be estimated by fitting a Poisson regression model. If there is over dispersion due to a high number of zero errors then a zero-inflated Poisson regression model will be used or an over dispersion parameter will be included into the model.

The mean difference in St Georges Respiratory Questionnaire, Modified MRC dyspnoea, PAMs and Hospital Anxiety and Depression between arms at the

final study visit will be estimated using analysis of covariance model including the baseline value of the score as well intervention arm as a covariate in the model. Time to exacerbation will be analysed in a similar manner to the two point increase in CAT score.

The number of adverse events (AE) and serious adverse events (SAE) will be tabulated by arm for each event and also by the number of patients reporting an event. No dictionary for coding adverse events will be used. Events will be recoded using terms of the clinical investigators choosing.

A full statistical analysis plan will be drawn up and agreed by the PI and study team prior to the final data extraction.

Ethical Review and Informed Consent

This study will be conducted in accordance with ICH GCP guidelines (directive CPMP/ICH/135/95), local regulatory requirements and the declaration of Helsinki, and all relevant local laws and regulations. The protocol and informed consent document will be reviewed and approved by a properly-constituted ethics committee. All aspects of the study will be explained in detail to prospective subjects and they will be informed of the voluntary nature of their participation. Written informed consent will be obtained from each subject using the approved documents, and each subject will receive a copy of his/her signed consents.

P.P.I

Members of Portsmouth and Southampton patient support group Breathe Easy reviewed the Patient Information Sheet (PIS) and informed consent form (ICF). Feedback was positive to the study and all who reviewed the study considered it a worthwhile research project and agreed if they had the opportunity to participate in the study they would.

Adverse Events

An adverse event (AE) is any unfavourable, harmful, or pathologic change in a research subject as indicated by physical signs, symptoms and/or clinically significant laboratory abnormalities.

This includes intercurrent illnesses, injuries, worsening of pre-existing conditions, and events. Stable pre-existing conditions and/or elective procedures to address them are not adverse events. Clinical laboratory findings are considered to be adverse events if regarded as clinically significant by the Investigator, or if these cause (or should have caused) a further diagnostic evaluation, or institution of any therapy.

All events fulfilling any part of the AE definition must be recorded in the source documents and on the adverse event CRF.

Note signs and symptoms of COPD in this study will not routinely be considered to be adverse events unless considered to be so by the PI.

Serious Adverse Events

A serious adverse event (SAE) is any adverse event that results in any of the following outcomes: (a) death; (b) an immediate threat to life; (c) inpatient hospitalization or prolongation of an existing hospitalization; (d) persistent or significant disability/incapacity; or (e) a congenital anomaly/birth defect.

Important medical events that do not result in one of these outcomes, but, based on appropriate medical judgment and that are deemed to jeopardize the subject or require medical or surgical intervention to avert one of the listed outcomes, may also be considered SAEs.

AE and SAE Exemptions

AE's and SAE's exempt from reporting because it is considered to be part of the expected change in COPD control or an exacerbation or in the judgement of the PI is clinically insignificantly to the patient (i.e Has not caused them harm)

Adverse events exempt from reporting include:

- Increase in rescue medication
- Additional course of steroids and or antibiotics for COPD exacerbation
- Increased unscheduled Healthcare usage (GP and Ed visits for deterioration in COPD control)
- Time off work for due to worsening COPD control
- Hospitalisation due to COPD exacerbation
- Increase in the number or intensity of COPD exacerbations.

- **References**

1 NICE (2010). National guidelines for the management of COPD in adults in primary and secondary care. Available at https://www.nice.org.uk/guidance/cg101 Accessed on 7th October 2014.

2 Department of Health (2012) An Outcomes Strategy for COPD and Asthma: NHS Companion Document. Available at: https://www.gov.uk/government/uploads/system/uploads/attachment_data/file/216531/dh_134001.pdf Accessed on 7th October 2014.

3 Consultation on a strategy for services for chronic obstructive pulmonary disease (COPD) in England. Available at http://www.dh.gov.uk Date accessed 06/10/14

4 National Institute for Health and Care Institute (2011) guidance.nice.org.uk (on line) available from: http://guidance.nice.org.uk/QS10 Date accessed 06/10/14

5 Reduction of hospital utilisation in patients with Chronic Obstructive Pulmonary Disease, A specific self management intervention. Bourbeau,J Julien. M, Maltasis, F et al 2003 Arch Intern Med 163 (5) 585-591.

6 Rice KL, Dewan N, Bloomfield HE, Grill J, Schult TM, Nelson DB et al. 317 Disease Management Program for Chronic Obstructive Pulmonary Disease: A 318 Randomized Controlled Trial. Am J Respir Crit Care Med 2010;Vol 182:890-319 896.

7 Patient Adherence in COPD. J Bourbeau, SJ Bartlett. Thorax 2008; 63, 831-383

8 M.North,T.Wilkinson.S.Bourne. The impact of an electronic self management system for patients with COPD. Eur Pespir J 2014; 44: Suppl. 58, 1413

9 P.W. Jones, G. Harding, P. Berry, I. Wiklund, W-H. Chen and N. Kline Leidy. Development and first validation of the COPD Assessment Test. Eur Respir J 2009, 34: 648–654.

10 Fletcher CM (Chairman). Standardised questionnaire on respiratory symptoms: a statement prepared and approved by the MRC Committee on the Aetiology of Chronic Bronchitis (MRC breathlessness score). BMJ 1960;2: 1665.

11 Jones PW, Quirk FH, Baveystock CM. The St George's Respiratory Questionnaire. Respir Med 1991;85(Suppl B):25-31.

12 Zigmond, ASSnaith, RP. The Hospital Anxiety and Depression Scale. *Acta Psychiatr Scand*. 1983;67:361–370.

13 Hibbard JH, Stockard J, Mahoney ER, Tusler M. Development of the patient activation measure (PAM): conceptualizing and measuring activation in patients and consumers. Health Serv Res.2004;39:1005–26.

14 Reilly MC, Zbrozek AS, Dukes EM. The validity and reproducibility of a work productivity and activity impairment instrument. *Pharmaco economics.* 1993;4:353–365.

15 McAuley,P Et al. Evaluation of a specific activity questionnaire to predict mortality in men referred for exercise testing. Am Heart J. 2006 Apr;151(4):890.e1-7

16 Browne, R. H. On the use of a pilot sample for sample size determination. Statistics in Medicine 14, 1933-1940 (1995).

17 Lancaster GA, Dodd S, Williamson PR. Design and analysis of pilot studies: recommendations for good practice. J Eval Clin Practice 2004;10:307-312
Browne RH. On the use of a pilot sample for sample size determination. Stat Med 1995;14:1933-1940

18 Sim J, Lewis M. The size of a pilot study for a clinical trial should be calculated in relation to considerations of precision and efficiency. J Clin Epidemiol 2012;65:301-308

19 Julious SA. Sample size of 12 per group rule of thumb for a pilot study. Pharm Stat 2005;4:287-291

Appendix 1: Study Schedule

	Case Identification<br>Day 1	Screening<br>Visit 1<br>(+- 7 days)	Telephone Contact		End of Study<br>Visit 4<br>90 days (+- 3 days)
			Visit 2 30 days (+- 3 days)	Visit 3 60 days (+- 3 days)	
Patient Information Sheet	✔				
Informed Consent		✔			
Verbal Consent			✔	✔	✔
Demographics		✔			
GP Letter		✔			
Medical History		✔			✔
CAT Questionnaire		✔	✔	✔	✔
SGRQ		✔			✔
Modified MRC		✔			✔
PAM Questionnaire		✔			✔
HAD Scale		✔			✔
WPAI Questionnaire		✔			✔
VSAQ score		✔			✔
Inhaler Technique Video		✔			✔
Inhaler technique Critical error sheet		✔			✔
Exacerbation History		✔			✔
Admission History		✔			✔
Randomisation		✔			
Health care usage			✔	✔	✔
Adverse and Serious Adverse Event reporting			✔	✔	✔
Patient feedback					✔

Appendix 2: PROMS

St Georges Respiratory Questionnaire

ST. GEORGE'S RESPIRATORY QUESTIONNAIRE
ORIGINAL ENGLISH VERSION

ST. GEORGE'S RESPIRATORY QUESTIONNAIRE (SGRQ)

This questionnaire is designed to help us learn much more about how your breathing is troubling you and how it affects your life. We are using it to find out which aspects of your illness cause you most problems, rather than what the doctors and nurses think your problems are.

Please read the instructions carefully and ask if you do not understand anything. Do not spend too long deciding about your answers.

Before completing the rest of the questionnaire:

	Very good	Good	Fair	Poor	Very poor
Please tick in one box to show how you describe your current health:	☐	☐	☐	☐	☐

Copyright reserved
P.W. Jones, PhD FRCP
Professor of Respiratory Medicine,
St. George's University of London,
Jenner Wing,
Cranmer Terrace,
London SW17 ORE, UK.

Tel. +44 (0) 20 8725 5371
Fax +44 (0) 20 8725 5955

UK/ English (original) version 1

continued…

f:\institut\cultadap\project\gsk1881\question\final versions\sgrqorig.doc 14/03/03

St. George's Respiratory Questionnaire
PART 1

Questions about how much chest trouble you have had over the past 3 months.

Please tick (✓) *one box for each question*:

	most days a week	several days a week	a few days a month	only with chest infections	not at all
1. Over the past 3 months, I have coughed:	☐	☐	☐	☐	☐
2. Over the past 3 months, I have brought up phlegm (sputum):	☐	☐	☐	☐	☐
3. Over the past 3 months, I have had shortness of breath:	☐	☐	☐	☐	☐
4. Over the past 3 months, I have had attacks of wheezing:	☐	☐	☐	☐	☐

5. During the past 3 months how many severe or very unpleasant attacks of chest trouble have you had?

   Please tick (✓) *one*:
   - more than 3 attacks ☐
   - 3 attacks ☐
   - 2 attacks ☐
   - 1 attack ☐
   - no attacks ☐

6. How long did the worst attack of chest trouble last?
   (Go to question 7 if you had no severe attacks)

   Please tick (✓) *one*:
   - a week or more ☐
   - 3 or more days ☐
   - 1 or 2 days ☐
   - less than a day ☐

7. Over the past 3 months, in an average week, how many good days (with little chest trouble) have you had?

   Please tick (✓) *one*:
   - No good days ☐
   - 1 or 2 good days ☐
   - 3 or 4 good days ☐
   - nearly every day is good ☐
   - every day is good ☐

8. If you have a wheeze, is it worse in the morning?

   Please tick (✓) *one*:
   - No ☐
   - Yes ☐

UK/ English (original) version 2

continued…

f:\institut\cultadap\project\gsk1881\question\final versions\sgrqoriq.doc 14/03/03

St. George's Respiratory Questionnaire
PART 2

Section 1

How would you describe your chest condition?

Please tick (✓) *one*:

- ☐ The most important problem I have
- ☐ Causes me quite a lot of problems
- ☐ Causes me a few problems
- ☐ Causes no problem

If you have ever had paid employment.

Please tick (✓) *one*:

- ☐ My chest trouble made me stop work altogether
- ☐ My chest trouble interferes with my work or made me change my work
- ☐ My chest trouble does not affect my work

Section 2

Questions about what activities usually make you feel breathless these days.

Please tick (✓) in ***each box*** that applies to you ***these days***:

	True	False
Sitting or lying still	☐	☐
Getting washed or dressed	☐	☐
Walking around the home	☐	☐
Walking outside on the level	☐	☐
Walking up a flight of stairs	☐	☐
Walking up hills	☐	☐
Playing sports or games	☐	☐

UK/ English (original) version 3

continued…

f:\institut\cultadap\project\gsk1881\question\final versions\sgrqorig.doc 14/03/03

St. George's Respiratory Questionnaire
PART 2

Section 3

Some more questions about your cough and breathlessness <u>these days</u>.

Please tick (✓) in ***each box*** that applies to you ***these days***:

	True	False
My cough hurts	☐	☐
My cough makes me tired	☐	☐
I am breathless when I talk	☐	☐
I am breathless when I bend over	☐	☐
My cough or breathing disturbs my sleep	☐	☐
I get exhausted easily	☐	☐

Section 4

Questions about other effects that your chest trouble may have on you <u>these days</u>.

Please tick (✓) in ***each box*** that applies to you ***these days***:

	True	False
My cough or breathing is embarrassing in public	☐	☐
My chest trouble is a nuisance to my family, friends or neighbours	☐	☐
I get afraid or panic when I cannot get my breath	☐	☐
I feel that I am not in control of my chest problem	☐	☐
I do not expect my chest to get any better	☐	☐
I have become frail or an invalid because of my chest	☐	☐
Exercise is not safe for me	☐	☐
Everything seems too much of an effort	☐	☐

Section 5

Questions about your medication, if you are receiving no medication go straight to section 6.

Please tick (✓) in ***each box*** that applies to you ***these days***:

	True	False
My medication does not help me very much	☐	☐
I get embarrassed using my medication in public	☐	☐
I have unpleasant side effects from my medication	☐	☐
My medication interferes with my life a lot	☐	☐

UK/ English (original) version 4

continued…

f:\institut\cultadap\project\gsk1881\question\final versions\sgrqoriq.doc 14/03/03

St. George's Respiratory Questionnaire
PART 2

Section 6

These are questions about how your activities might be affected by your breathing.

Please tick (✓) in *each box* that applies to you *because of your breathing*:

	True	False
I take a long time to get washed or dressed	☐	☐
I cannot take a bath or shower, or I take a long time	☐	☐
I walk slower than other people, or I stop for rests	☐	☐
Jobs such as housework take a long time, or I have to stop for rests	☐	☐
If I walk up one flight of stairs, I have to go slowly or stop	☐	☐
If I hurry or walk fast, I have to stop or slow down	☐	☐
My breathing makes it difficult to do things such as walk up hills, carrying things up stairs, light gardening such as weeding, dance, play bowls or play golf	☐	☐
My breathing makes it difficult to do things such as carry heavy loads, dig the garden or shovel snow, jog or walk at 5 miles per hour, play tennis or swim	☐	☐
My breathing makes it difficult to do things such as very heavy manual work, run, cycle, swim fast or play competitive sports	☐	☐

Section 7

We would like to know how your chest <u>usually</u> affects your daily life.

Please tick (✓) in *each box* that applies to you *because of your chest trouble*:

	True	False
I cannot play sports or games	☐	☐
I cannot go out for entertainment or recreation	☐	☐
I cannot go out of the house to do the shopping	☐	☐
I cannot do housework	☐	☐
I cannot move far from my bed or chair	☐	☐

St. George's Respiratory Questionnaire

Here is a list of other activities that your chest trouble may prevent you doing. (You do not have to tick these, they are just to remind you of ways in which your breathlessness may affect you):

Going for walks or walking the dog
Doing things at home or in the garden
Sexual intercourse
Going out to church, pub, club or place of entertainment
Going out in bad weather or into smoky rooms
Visiting family or friends or playing with children

Please write in any other important activities that your chest trouble may stop you doing:

....
....
....
....

Now would you tick in the box (one only) which you think best describes how your chest affects you:

- It does not stop me doing anything I would like to do ☐
- It stops me doing one or two things I would like to do ☐
- It stops me doing most of the things I would like to do ☐
- It stops me doing everything I would like to do ☐

Thank you for filling in this questionnaire. Before you finish would you please check to see that you have answered all the questions.

COPD Assessment Test

Your name:

Today's date:

How is your COPD? Take the COPD Assessment Test™ (CAT)

This questionnaire will help you and your healthcare professional measure the impact COPD (Chronic Obstructive Pulmonary Disease) is having on your wellbeing and daily life. Your answers, and test score, can be used by you and your healthcare professional to help improve the management of your COPD and get the greatest benefit from treatment.

For each item below, place a mark (X) in the box that best describes you currently. Be sure to only select one response for each question.

Example: I am very happy (0) (X) (2) (3) (4) (5) I am very sad

			SCORE
I never cough	0 1 2 3 4 5	I cough all the time	
I have no phlegm (mucus) in my chest at all	0 1 2 3 4 5	My chest is completely full of phlegm (mucus)	
My chest does not feel tight at all	0 1 2 3 4 5	My chest feels very tight	
When I walk up a hill or one flight of stairs I am not breathless	0 1 2 3 4 5	When I walk up a hill or one flight of stairs I am very breathless	
I am not limited doing any activities at home	0 1 2 3 4 5	I am very limited doing activities at home	
I am confident leaving my home despite my lung condition	0 1 2 3 4 5	I am not at all confident leaving my home because of my lung condition	
I sleep soundly	0 1 2 3 4 5	I don't sleep soundly because of my lung condition	
I have lots of energy	0 1 2 3 4 5	I have no energy at all	
		**TOTAL SCORE**	

COPD Assessment Test and CAT logo is a trademark of the GlaxoSmithKline group of companies.
© 2009 GlaxoSmithKline. All rights reserved.

Modified MRC (mMRC) Dyspnoea Score

Modified MRC Dyspnoea Score

Grade		
0	I only get breathless with strenuous exercise.	
1	I get short of breath when hurrying on level ground or walking up a slight hill.	
2	On level ground, I walk slower than people of the same age because of breathlessness, or have to stop for breath when walking at my own pace.	
3	I stop for breath after walking about 100 yards or after a few minutes on level ground.	
4	I am too breathless to leave the house or I am breathless when dressing.	

Hospital Anxiety and Depression Test

Patient Name:
ID Number:
Date:

HAD Scale

This questionnaire is designed to help us know how you feel. Read each item and place a firm tick in the box opposite the reply, which comes closest to how you have been feeling in the past week.

Don't take too long over your replies; your immediate reaction to each item will probably be more accurate than a long thought-out response.

Tick one box only in each section

1 I feel tense or wound up:
- Most of the time (3) ☐
- A lot of the time (2) ☐
- Time to time, occasionally (1) ☐
- Not at all (0) ☐

2 I still enjoy the things I used to enjoy:
- Definitely as much (0) ☐
- Not quite so much (1) ☐
- Only a little (2) ☐
- Hardly at all (3) ☐

3 I get a sort of frightened feeling as if something awful is about to happen:
- Very definitely and quite badly (3) ☐
- Yes, but not too badly (2) ☐
- A little, but it doesn't worry me (1) ☐
- Not at all (0) ☐

4 I can laugh and see the funny side of things:
- As much as I always could (0) ☐
- Not quite so much now (1) ☐
- Definitely not so much now (2) ☐
- Not at all (3) ☐

5 Worrying thoughts go through my mind:
- A great deal of the time (3) ☐
- A lot of the time (2) ☐
- From time to time but not too often (1) ☐
- Only occasionally (0) ☐

6 I feel cheerful:
- Not at all (3) ☐
- Not often (2) ☐
- Sometimes (1) ☐
- Most of the time (0) ☐

7 I can sit at ease and feel relaxed:
- Definitely (0) ☐
- Usually (1) ☐
- Not often (2) ☐
- Not at all (3) ☐

8 I feel as if I am slowed down:
- Nearly all the time (3) ☐
- Very often (2) ☐
- Sometimes (1) ☐
- Not at all (0) ☐

9 I get a sort of frightened feeling like "butterflies" in the stomach:
- Not at all (0) ☐
- Occasionally (1) ☐
- Quite often (2) ☐
- Very often (3) ☐

10 I have lost interest in my appearance:
- Definitely (3) ☐
- I don't take so much care as I should (2) ☐
- I may not take quite as much care (1) ☐
- I take just as much care as ever (0) ☐

11 I feel restless as if I have to be on the move:
- Very much indeed (3) ☐
- Quite a lot (2) ☐
- Not very much (1) ☐
- Not at all (0) ☐

12 I look forward with enjoyment to things:
- As much as I ever did (0) ☐
- Rather less than I used to (1) ☐
- Definitely less than I used to (2) ☐
- Hardly at all (3) ☐

13 I get sudden feelings of panic:
- Very often indeed (3) ☐
- Quite often (2) ☐
- Not very often (1) ☐
- Not at all (0) ☐

14 I can enjoy a good book or radio or TV programme:
- Often (0) ☐
- Sometimes (1) ☐
- Not often (2) ☐
- Very seldom (3) ☐

ANXIETY SCORE DEPRESSION SCORE

Patient Activation Measurement (PAM)

Below are some statements that people sometimes make when they talk about their health. Please indicate how much you agree or disagree with each statement as it applies to you personally by circling your answer. Your answers should be what is true for you and not just what you think others want you to say.

If the statement does not apply to you, circle N/A.

1. When all is said and done, I am the person who is responsible for taking care of my health	Disagree Strongly	Disagree	Agree	Agree Strongly	N/A
2. Taking an active role in my own health care is the most important thing that affects my health	Disagree Strongly	Disagree	Agree	Agree Strongly	N/A
3. I am confident I can help prevent or reduce problems associated with my health	Disagree Strongly	Disagree	Agree	Agree Strongly	N/A
4. I know what each of my prescribed medications do	Disagree Strongly	Disagree	Agree	Agree Strongly	N/A
5. I am confident that I can tell whether I need to go to the doctor or whether I can take care of a health problem myself	Disagree Strongly	Disagree	Agree	Agree Strongly	N/A
6. I am confident that I can tell a doctor concerns I have even when he or she does not ask	Disagree Strongly	Disagree	Agree	Agree Strongly	N/A
7. I am confident that I can follow through on medical treatments I may need to do at home	Disagree Strongly	Disagree	Agree	Agree Strongly	N/A
8. I understand my health problems and what causes them	Disagree Strongly	Disagree	Agree	Agree Strongly	N/A
9. I know what treatments are available for my health problems	Disagree Strongly	Disagree	Agree	Agree Strongly	N/A
10. I have been able to maintain (keep up with) lifestyle changes, like eating right or exercising	Disagree Strongly	Disagree	Agree	Agree Strongly	N/A
11. I know how to prevent problems with my health	Disagree Strongly	Disagree	Agree	Agree Strongly	N/A
12. I am confident I can figure out solutions when new problems arise with my health	Disagree Strongly	Disagree	Agree	Agree Strongly	N/A
13. I am confident that I can maintain lifestyle changes, like eating right and exercising, even during times of stress	Disagree Strongly	Disagree	Agree	Agree Strongly	N/A

Insignia Health. "Patient Activation Measure; Copyright © 2003-2011, University of Oregon. All Rights reserved."
Contact Insignia Health at www.insigniahealth.com

Insignia Health, LLC □ Proprietary and Confidential □ © 2011 □ For use with a valid copyright license only

Work Productivity Activity Impairment Questionnaire

Work Productivity and Activity Impairment Questionnaire:
General Health V2.0 (WPAI:GH)

The following questions ask about the effect of your health problems on your ability to work and perform regular activities. By health problems we mean any physical or emotional problem or symptom. *Please fill in the blanks or circle a number, as indicated.*

1. Are you currently employed (working for pay)? ____ NO ____ YES
   If NO, check "NO" and skip to question 6.

The next questions are about the **past seven days**, not including today.

2. During the past seven days, how many hours did you miss from work because of your COPD? *Include hours you missed on sick days, times you went in late, left early, etc., because of your health problems. Do not include time you missed to participate in this study.*

   _____HOURS

3. During the past seven days, how many hours did you miss from work because of any other reason, such as vacation, holidays, time off to participate in this study?

   _____HOURS

4. During the past seven days, how many hours did you actually work?

   _____HOURS *(If "0", skip to question 6.)*

5. During the past seven days, how much did your COPD affect your productivity while you were working?

Think about days you were limited in the amount or kind of work you could do, days you accomplished less than you would like, or days you could not do your work as carefully as usual. If health problems affected your work only a little, choose a low number. Choose a high number if health problems affected your work a great deal.

Consider only how much COPD affected productivity while you were working.

Health problems had no effect on my work	0	1	2	3	4	5	6	7	8	9	10	Health problems completely prevented me from working

CIRCLE A NUMBER

6. During the past seven days, how much did your COPD affect your ability to do your regular daily activities, other than work at a job?

By regular activities, we mean the usual activities you do, such as work around the house, shopping, childcare, exercising, studying, etc. Think about times you were limited in the amount or kind of activities you could do and times you accomplished less than you would like. If health problems affected your activities only a little, choose a low number. Choose a high number if health problems affected your activities a great deal.

Consider only how much COPD affected your ability to do your regular daily activities, other than work at a job.

Health problems had no effect on my daily activities	0	1	2	3	4	5	6	7	8	9	10	Health problems completely prevented me from doing my daily activities

CIRCLE A NUMBER

Veterans Specific Activity Questionnaire (VSAQ)

The following is a list of activities that increase in difficulty as you read down the page. Think carefully, then underline the first activity that, if you performed it for a period of time, would typically cause fatigue, shortness of breath, chest discomfort, or otherwise cause you to want to stop. If you do not normally perform a particular activity, try to imagine what it would be like if you did.

1 METs	Eating, getting dressed, working at a desk
2 METs	Taking a shower, shopping, cooking, walking down 8 steps
3 METs	Walking slowly on a flat surface for 1 or 2 blocks, a moderate amount of work around the house, such as vacuuming, sweeping the floors, or carrying groceries
4 METs	Light garden work (ie, raking leaves, weeding, sweeping, or pushing a power mower), painting, or light carpentry
5 METs	Walking briskly, social dancing, washing the car
6 METs	Play 9 holes of golf carrying your own clubs. Heavy carpentry, mow lawn with push mower
7 METs	Carrying 60 pounds, perform heavy outdoor work (ie, digging, spading soil, etc), walking uphill
8 METs	Carrying groceries upstairs, move heavy furniture, jog slowly on flat surface, climb stairs quickly
9 METs	Cycling at a moderate pace, sawing wood, skipping (slowly)
10 METs	Brisk swimming, cycling up a hill, jog 6 miles per hour
11 METs	Carry a heavy load (ie, a child or firewood) up 2 flights of stairs, cross-country ski, bicycling briskly, continuously
12 METs	Running briskly, continuously (level ground, 8 min per mile)
13 METs	Any competitive activity, including those that involve intermittent sprinting, running competitively, rowing competitively, bicycle riding

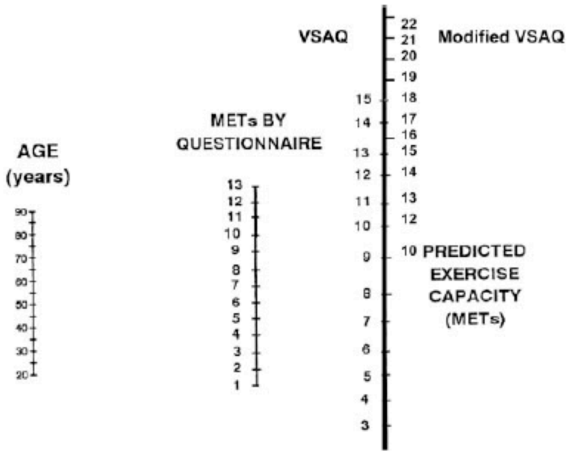
VSAQ
Modified VSAQ
METs BY QUESTIONNAIRE
AGE (years)
PREDICTED EXERCISE CAPACITY (METs)

Appendix 3: Interventions- My COPD and Written Plan

Screen Shots of the web based self management solution 'myCOPD'

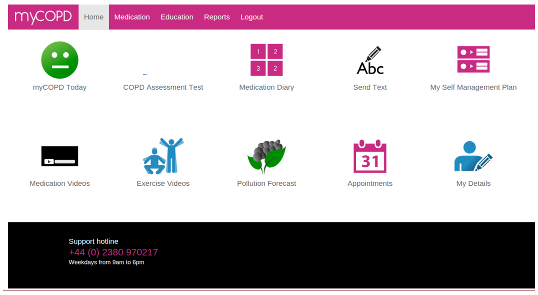

Home page. Patients can choose from a selection of icons to select different activities

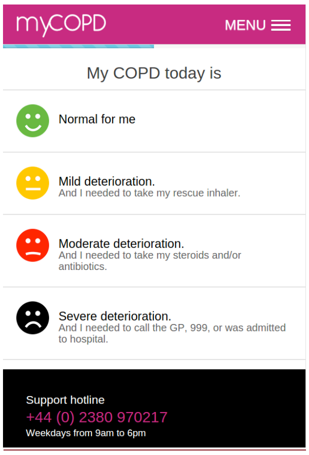

Patients can record daily how they are feeling and complete a symptom questionnaire daily. This data is then produced in a graph format creating a timeline of data for the patient

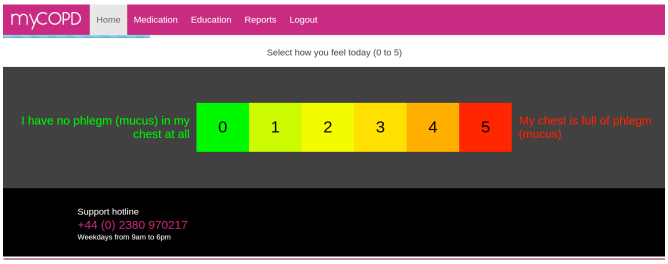

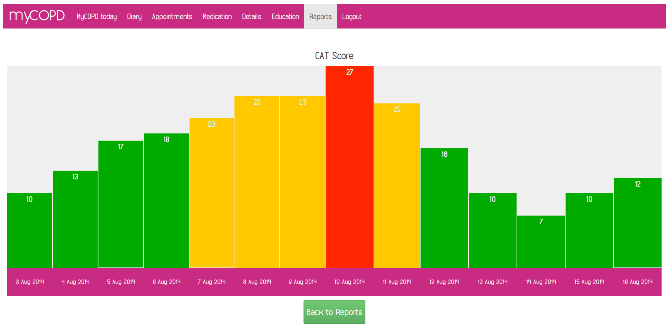

Patient will be able to access Self management advice from the website which gives clear instruction on what to do if their condition deteriorates.

Mild deterioration

I am more breathless than normal but have no fevers or change in sputum colour or volume.

When I have mild deterioration

Please continue your regular treatment and use reliever inhaler as prescribed.

Moderate deterioration

I am more breathless than normal and coughing up more sputum or sputum has changed in colour. Or I am much more breathless despite taking my reliever medication.

I have a moderate deterioration in my symptoms

Please continue to use your medication and

- **Your antibiotics** (As prescribed and advised by your doctor)
- **Prednisolone** (As prescribed and advised by your doctor)

Tell your COPD nurse or surgery within 2 days of starting this treatment.

Severe deterioration

My breathing is much worse than normal despite treatment or I have chest pain and/or high fevers.

I am much worse than normal

Please call your GP the same day or call 999 if you are too unwell to wait to see your doctor.

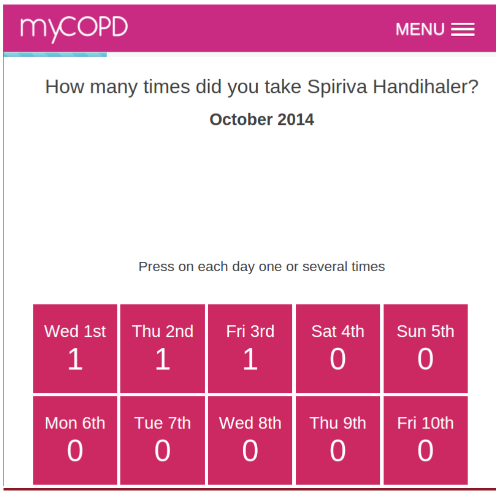

Image of the medication diary

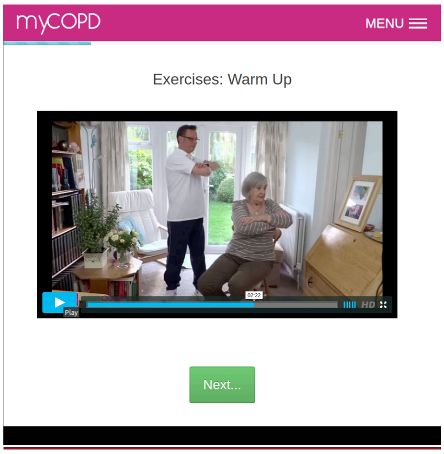

Bite size Pulmonary Rehabilitation videos to promote healthy exercise in the home

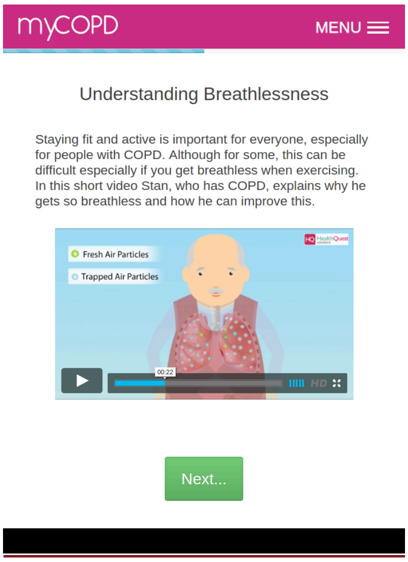

Education videos designed to improve patient's knowledge of different aspects of COPD

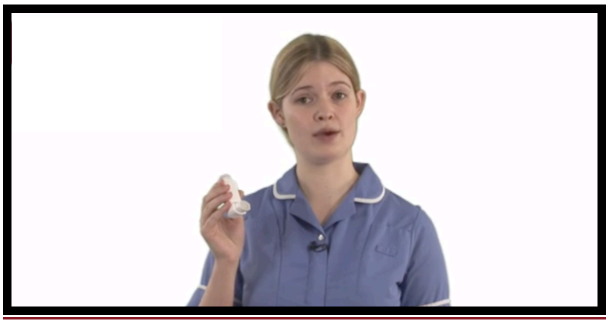

Instructional inhaler videos

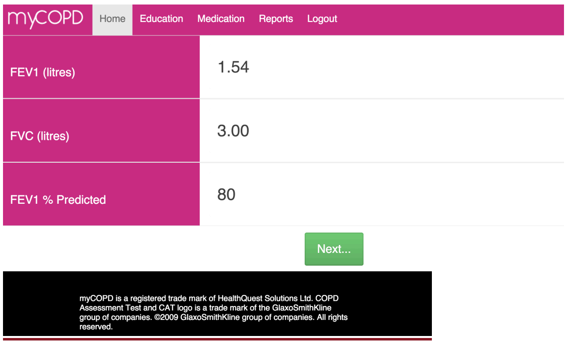

My Details allows the patient to input details about their COPD from next of kin and GP details to lung function data

Deleted: n

Written Self Management Plan

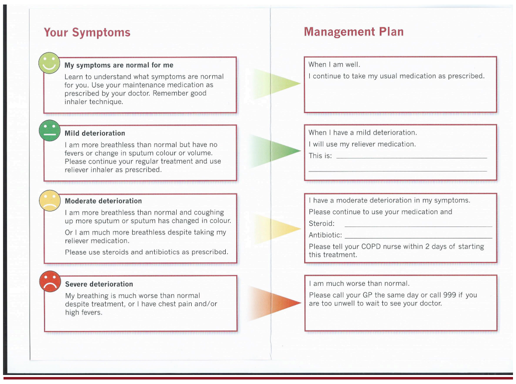

Your Symptoms

My symptoms are normal for me
Learn to understand what symptoms are normal for you. Use your maintenance medication as prescribed by your doctor. Remember good inhaler technique.

Mild deterioration
I am more breathless than normal but have no fevers or change in sputum colour or volume. Please continue your regular treatment and use reliever inhaler as prescribed.

Moderate deterioration
I am more breathless than normal and coughing up more sputum or sputum has changed in colour.
Or I am much more breathless despite taking my reliever medication.
Please use steroids and antibiotics as prescribed.

Severe deterioration
My breathing is much worse than normal despite treatment, or I have chest pain and/or high fevers.

Management Plan

When I am well.
I continue to take my usual medication as prescribed.

When I have a mild deterioration.
I will use my reliever medication.
This is: ____________

I have a moderate deterioration in my symptoms.
Please continue to use your medication and
Steroid: ____________
Antibiotic: ____________
Please tell your COPD nurse within 2 days of starting this treatment.

I am much worse than normal.
Please call your GP the same day or call 999 if you are too unwell to wait to see your doctor.

Printed Self management action plan

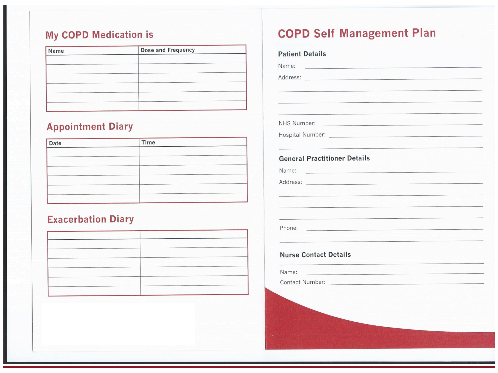

My COPD Medication is

Name	Dose and Frequency

Appointment Diary

Date	Time

Exacerbation Diary

COPD Self Management Plan

Patient Details
Name: ____________
Address: ____________

NHS Number: ____________
Hospital Number: ____________

General Practitioner Details
Name: ____________
Address: ____________

Phone: ____________

Nurse Contact Details
Name: ____________
Contact Number: ____________

Patient details section of the self management plan

Subject Initials _ _ _

Subject Number _ _ _

Visit One / Two

Date _ _ / _ _ / _ _ _ _

**Meter Dose Inhaler (MDI)**	YES	NO
Remove cap		
Shake well		
Breathe out normally		
Keep head upright or slightly tilted		
Seal lips around mouthpiece		
Inhale slowly, actuating once during first half of inhalation		
Continue slow and deep inhalation		
Exhale after holding breath for 5 or more seconds		
Any other comments		

Subject Initials _ _ _

Subject Number _ _ _

Visit One / Two

Date _ _ / _ _ / _ _ _ _

**MDI With Spacer**	YES	NO
Remove caps		
Shake MDI well		
Insert MDI into spacer		
Breathe out normally		
Seal lips around mouthpiece		
Actuate MDI		
Inhale slowly and deeply		
Exhale after holding breath for 5 or more seconds		
Any other comments		

Subject Initials _ _ _

Subject Number _ _ _

Visit One / Two

Date _ _ / _ _ / _ _ _ _

**Turbuhaler**	YES	NO
Hold upright without occluding air vents		
Turn coloured wheel one way, then back		
Breathe out normally and away from mouthpiece		
Seal lips around mouthpiece without occluding air vents		
Inhale forcefully and deeply		
Hold breath for at least 5 seconds		
Exhale but not through inhaler		
Any Other comments		

Subject Initials _ _ _

Subject Number _ _ _

Visit One / Two

Date _ _ / _ _ / _ _ _ _

**Handihaler**	YES	NO
Open lid and mouthpiece		
Place capsule in chamber		
Close mouthpiece, ensuring click is heard		
Holding inhaler upright, press blue button fully		
Breathe out normally and away from inhaler		
Seal lips around mouthpiece		
Inhale forcefully and deeply so that capsule vibrates		
Exhaled after holding breath for 5 or more seconds		
Any other comments		

Subject Initials _ _ _

Subject Number _ _ _

Visit One / Two

Date _ _ / _ _ / _ _ _ _

**Ellipta Inhaler**	YES	NO
Open to expose mouthpiece		
Slide lever until click heard		
Breathe out normally and away from inhaler		
Seal lips around mouthpiece		
Inhale forcefully and deeply		
Hold breath for 4-5 seconds		
Exhale but not through inhaler		
Any other comments		

Subject Initials _ _ _

Subject Number _ _ _

Visit One / Two

Date _ _ / _ _ / _ _ _ _

**Respimat**	YES	No
Holds inhaler in upright position		
Turn the base in a anticlockwise direction ensuring click sound is heard		
Open the protective cap to expose the mouthpiece		
Breath out normally away from inhaler		
Place lips around mouthpiece without covering the air vents		
Ensure the inhaler is in a horizontal position		
Inhale slowly and deeply actuating once during the first half of inhalation.		
Exhaled after holding breath for 5 seconds or longer		
Any other comments		

Subject Initials _ _ _

Subject Number _ _ _

Visit One / Two

Date _ _ / _ _ / _ _ _ _

**Nexthaler**	YES	NO
Hold inhaler in a upright position		
Open to expose mouthpiece		
Breathe out normally away from mouthpiece		
Seal lips around mouthpiece		
Inhale forcefully and deeply		
Hold breathe for 5 seconds or more		
Exhale but not through the inhaler		
Any other comments		

Subject Initials _ _ _

Subject Number _ _ _

Visit One / Two

Date _ _ / _ _ / _ _ _ _

**Easyhaler**	YES	NO
Remove protective cap		
Shake inhaler well		
Breathe out normally away from the mouthpiece		
Seal lips around mouthpiece		
Inhale forcefully and deeply		
Exhaled after Holding breathe for 5 seconds or more.		
Any other comments		

Subject Initials _ _ _

Subject Number _ _ _

Visit One / Two

Date _ _ / _ _ / _ _ _ _

**Easibreathe**	YES	NO
Shake inhaler well		
Open Cap		
Breathe out normally away from inhaler		
Seal lips around mouthpiece		
Inhale steadily a ‘puff’ sound will indicate actuation		
Exhaled after Holding breathe for 5 seconds or more		
Any other comments		

Subject Initials _ _ _

Subject Number _ _ _

Visit One / Two

Date _ _ / _ _ / _ _ _ _

**Autohaler**	YES	NO
Remove mouthpiece cap		
Shake inhaler well		
Pull up lever on top of the inhaler		
Breathe out normally away from inhaler		
Seal lips around mouthpiece		
Inhale slowly and deeply		
Exhale after holding breathe for 5 seconds or more		
Any other comments		

Subject Initials _ _ _

Subject Number _ _ _

Visit One / Two

Date _ _ / _ _ / _ _ _ _

**Breezehaler**		
Remove cap		
Tilt mouthpiece		
Remove capsule and place in chamber		
Close mouthpiece ensuring click is heard		
Holding inhaler in upright position press coloured buttons on the side to pierce capsule		
Breathe out normally away from inhaler		
Seal lips around mouthpiece		
Inhale forcefully and deeply. A whirring sound should be heard		
Hold breathe for 5 seconds or more		
Exhale fully		
Any other comments		

Subject Initials _ _ _

Subject Number _ _ _

Visit One / Two

Date _ _ / _ _ / _ _ _ _

**Spiromax Inhaler**	YES	NO
Do not shake inhaler		
Hold inhaler upright		
Open cap until a 'Click' is heard		
Do not cover airvents		
Inhale strongly and deeply		
Hold breathe for 5 seconds or more		
Exhale away from inhaler		
Replace cap		
Any other comments		

Subject Initials _ _ _

Subject Number _ _ _

Visit One / Two

Date _ _ / _ _ / _ _ _ _

**Genuair Inhaler**	YES	NO
Do not shake inhaler		
Hold inhaler horizontally with the green button facing up		
Remove protective cap		
Press green button and release		
Coloured control window turns to green		
Breathe out normally and away from inhaler		
Seal lips around mouthpiece		
Inhale forcefully and deeply		
Exhaled after Holding breath for 4-5 seconds		
Any other Comments		

Subject Initials _ _ _

Subject Number _ _ _

Visit One / Two

Date _ _ / _ _ / _ _ _ _

**Accuhaler**		
Holding inhaler in a horizontal position Expose mouth piece		
Keeping in horizontal position slide lever		
Breathe out normally away from		
Seal lips around mouthpiece		
Breath in steadily and deeply		
Remove inhaler from mouth		
Breath hold for 5-10 seconds		
Exhale slowly		
Close accuhaler		
Any other comments		

Supplementary Figure 1: Study protocol

Shown is the study protocol for the clinical study.

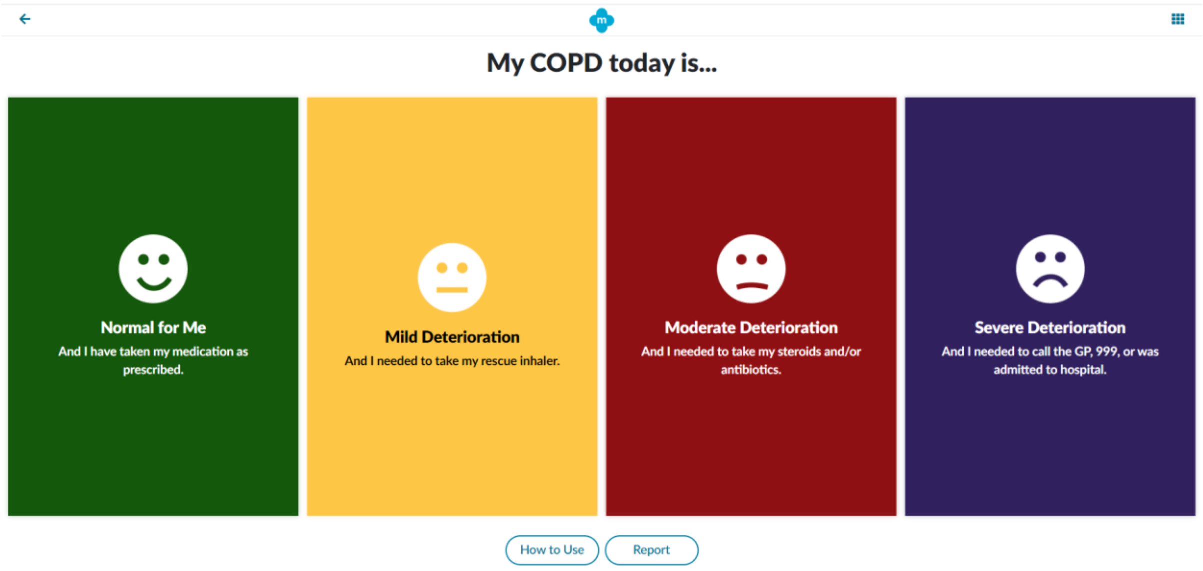

*Note: Patients are required to click on how they are feeling daily. This is recorded and interpreted into a graph format to observe symptom trends.

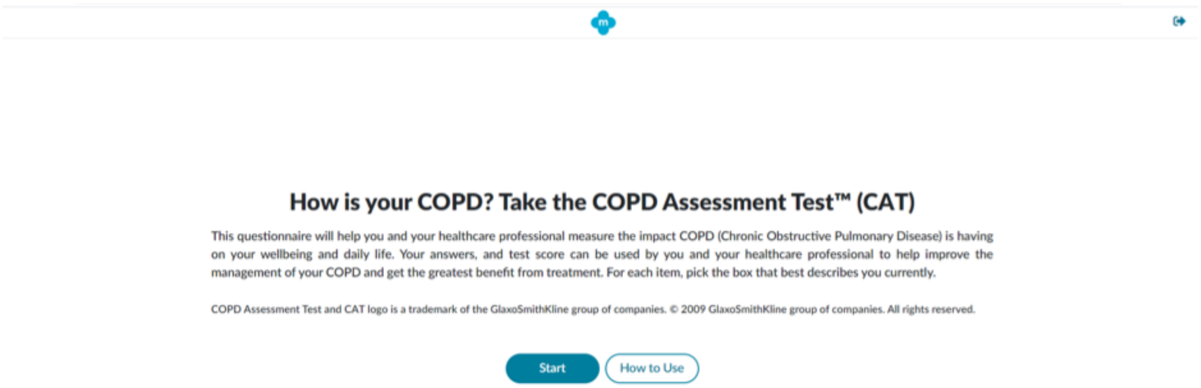

*Note: On first access and every four weeks patients will need to complete CAT

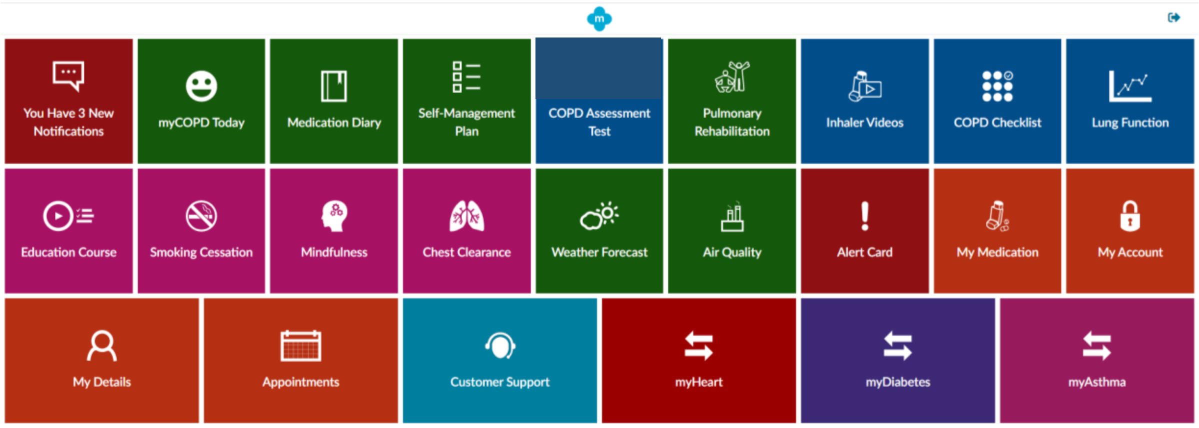

*Note: myCOPD app dashboard displaying tiles for usage. Green = daily or every other day use, Blue = every few weeks, Pink = educational, Orange = action, Red = notifications

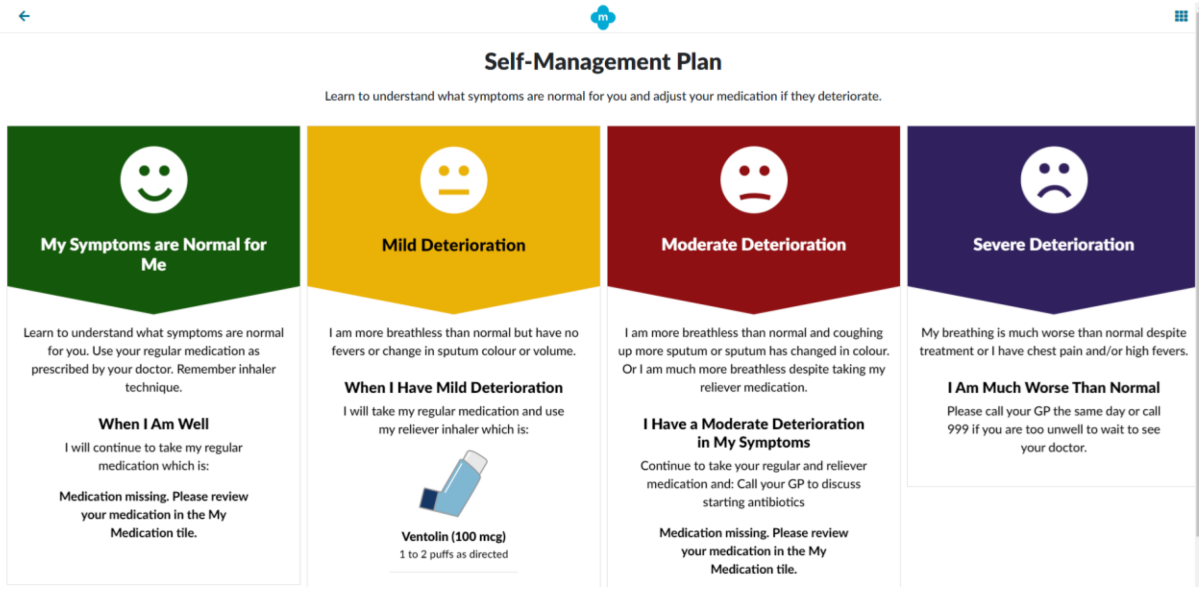

*Note: Self-Management Plan example

Supplementary Figure 2: myCOPD app content

Indicated is some of the app screenshots with colour coded tiles with images to indicate their content.

Supplementary Table 1: Descriptions of missingness for effectiveness outcomes

Missing Data for Effectiveness Outcome	Total (N=41)	Treatment Groups TAU (n=21)	MyCOPD (n=20)
**COPD Assessment Test Score**			
Month 0	0 (0%)	0 (0%)	0 (0%)
Month 1	0 (0%)	0 (0%)	0 (0%)
Month 2	4 (9.8%)	2 (9.5%)	2 (10%)
Month 3	0 (0%)	0 (0%)	0 (0%)
**Modified MRC scale for Dyspnoea**			
Month 0	0 (0%)	0 (0%)	0 (0%)
Month 3	0 (0%)	0 (0%)	0 (0%)
**Patient Activation Measure (PAM)**			
Month 0	0 (0%)	0 (0%)	0 (0%)
Month 3	3 (7.3%)	2 (9.5%)	1 (5%)
**Hospital Anxiety and Depression scale (HAD)**			
Month 0	0 (0%)	0 (0%)	0 (0%)
Month 3	0 (0%)	0 (0%)	0 (0%)
**St Georges Respiratory Questionnaire (SGRQ)**			
Month 0	0 (0%)	0 (0%)	0 (0%)
Month 3	0 (0%)	0 (0%)	0 (0%)
**Work Productivity Activity Impairment Questionnaire**			
Month 0	0 (0%)	0 (0%)	0 (0%)
Month 3	0 (0%)	0 (0%)	0 (0%)
**Veterans Specific Activity Questionnaire (VSAQ) Score**			
Month 0	0 (0%)	0 (0%)	0 (0%)
Month 3	0 (0%)	0 (0%)	0 (0%)
**Number of recorded Exacerbations**			
Month 0	0 (0%)	0 (0%)	0 (0%)
Month 3	0 (0%)	0 (0%)	0 (0%)

This table does not include the 6 subjects who withdrew from the study
